# Cell disorientation by loss of SHH-dependent mechanosensation causes cyclopia

**DOI:** 10.1126/sciadv.abn2330

**Published:** 2022-07-13

**Authors:** Daisuke Ohtsuka, Naoki Kida, Sang-Woo Lee, Naofumi Kawahira, Yoshihiro Morishita

**Affiliations:** ^1^ Laboratory for Developmental Morphogeometry, RIKEN Center for Biosystems Dynamics Research, Kobe 650-0047, Japan.; ^2^ Department of Molecular Cell Developmental Biology, School of Life Science, University of California, Los Angeles (UCLA), Los Angeles, CA 90095, USA.

## Abstract

The physical causes of organ malformation remain largely unclear in most cases due to a lack of information on tissue/cell dynamics. Here, we address this issue by considering onset of cyclopia in sonic hedgehog (SHH)–inhibited chick embryos. We show that ventral forebrain–specific self-organization ability driven by SHH-dependent polarized patterns in cell shape, phosphorylated myosin localization, and collective cell motion promotes optic vesicle elongation during normal development. Stress loading tests revealed that these polarized dynamics result from mechanical responses. In particular, stress and active tissue deformation satisfy orthogonality, defining an SHH-regulated morphogenetic law. Without SHH signaling, cells cannot detect the direction of stress and move randomly, leading to insufficient optic vesicle elongation and consequently a cyclopia phenotype. Since polarized tissue/cell dynamics are common in organogenesis, cell disorientation caused by loss of mechanosensation could be a pathogenic mechanism for other malformations.

## Teaser

SHH-deficient neuroepithelia lose mechanosensation and polarity in collective motion, resulting in a cyclopia phenotype.

## INTRODUCTION

In recent decades, genetics and molecular biology studies identified multiple morphogenetic genes essential for normal organ development. *Sonic hedgehog* (*SHH*) is one of such genes and known as a key regulator for morphogenesis of various organs and anatomical structures such as the brain and limbs during vertebrate embryonic development. Defects in *SHH* are responsible for different congenital malformations (*1*–*4*). Holoprosencephaly (HPE) is a typical example of a malformation associated with *SHH* gene deficiency, and severe cases of HPE have cyclopia (*5*). Previous studies using model animals on the pathogenic mechanisms of HPE phenotypes including cyclopia have focused on abnormalities in spatial patterning of SHH downstream gene expressions, induction of differentiation, and regulation of cell proliferation/apoptosis in the developing neuroepithelium (*6*–*8*). However, as with almost all other congenital malformations, the physical causes of cyclopia-like phenotypes in terms of tissue and cell dynamics including tissue deformation, collective cell motion, and their relevance to mechanical stress are unclear. We here address this issue by combining four-dimensional (4D) imaging, quantitative analysis of tissue/cell dynamics, and mechanical simulations.

## RESULTS

### Critical time window of SHH action

**Fig. 1.**
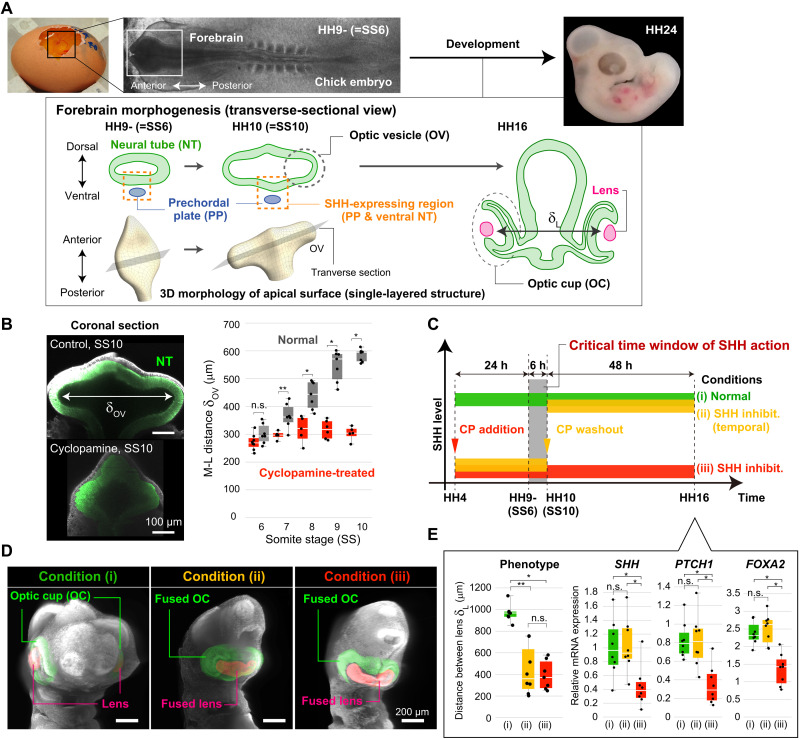
A critical time window for SHH action. (**A**) Normal forebrain development. During the OV elongation phase, SHH expression only in the ventral forebrain makes the region dorsoventrally asymmetric in terms of SHH signaling activity. (**B**) M-L distance between the left and right OV tips (δ_OV_) in the absence and presence of the SHH inhibitor cyclopamine (CP). (**C** to **E**) Phenotypes and expression levels of *SHH* and its downstream genes in normal development [condition (i)] and after temporal (ii) or continuous (iii) inhibition of SHH signaling; (C) SHH inhibition time course, (D) phenotype around HH16, and (E) distance between the centers of the left and right lenses (δ_L_) for both normal separated and abnormal fused cases (left), and mRNA expression levels of *SHH* and its downstream genes by qPCR assay (right). In (B) and (E), * and ** indicates *P* < 0.01 by Student’s (two-sided) and Welch’s *t* tests (two-sided), respectively. n.s., not significant.

In the normal embryo, forebrain morphogenesis begins with the formation of a tubular structure called the neural tube (NT) from the sheet-like neural plate (*9*). Next, a protrusion called the optic vesicle (OV) forms at the anterior part of the NT. The OV then elongates laterally, and later, an eye forms at its tip (Fig. 1A). In SHH-deficient embryos, early events during forebrain morphogenesis such as formation of the NT and OV primordia proceed normally, but subsequent OV elongation does not (Fig. 1, A and B) (*1*, *10*). We quantified the medio-lateral (M-L) distance between the left and right OV tips based on two-photon microscopic images of forebrain regions in chick embryos that were treated or not with the SHH inhibitor cyclopamine (Fig. 1B). We first confirmed that, even if SHH signaling is inhibited well before forebrain formation, early events including NT and OV primordium formation, which in chicks occurs by somite stage (SS) 6, are normal. However, with continued SHH inhibition, OV elongation was significantly suppressed, and a cyclopia-like phenotype was eventually obtained (Fig. 1, B to E, and fig. S1; see also fig. S2 for the similar results with other modes of SHH inhibition). Washout of cyclopamine at SS10, when wild-type OVs have elongated sufficiently for normal development, restored SHH signaling to normal levels but did not recover morphological abnormalities at later stages (e.g., HH16) (Fig. 1, C to E). In addition, when cyclopamine was added at SS10 (and not before), the morphology was only slightly affected (fig. S1C); no fusion of the left and right lenses was observed. These results demonstrate the existence of a critical time window, which in chicks is several hours between around SS6 and SS10, during which SHH action can substantially affect later morphogenesis. Inadequate OV elongation due to loss of SHH signaling during this time window can prevent separation between the left and right OV tips and cause a cyclopia-like malformation. Thus, we focused on the role of SHH in regulating tissue/cell dynamics during this critical time window.

Previously, we reconstructed tissue deformation maps and quantified the spatiotemporal patterns of local tissue deformation during the critical window for normal forebrain development (see also fig. S3 for a brief summary) (*11*). We showed that OV elongation (i) occurs independently of cell division and (ii) is driven by anisotropic tissue deformation involving directional tissue stretch along the M-L axis and anterior-posterior (A-P) shrinkage across the entire forebrain region and not just the tips of the OV. These findings indicate that growth-based morphogenetic mechanisms such as oriented cell division and/or differential growth are not core mechanisms for OV elongation and that, beyond regulation of cellular states in terms of differentiation and patterning, the relationship between SHH signaling and polarized cell dynamics that induces tissue-level anisotropic deformation is key to elucidating critical pathogenic mechanisms of cyclopia in SHH-deficient embryos.

### SHH regulates A-P–oriented cell polarity

**Fig. 2.**
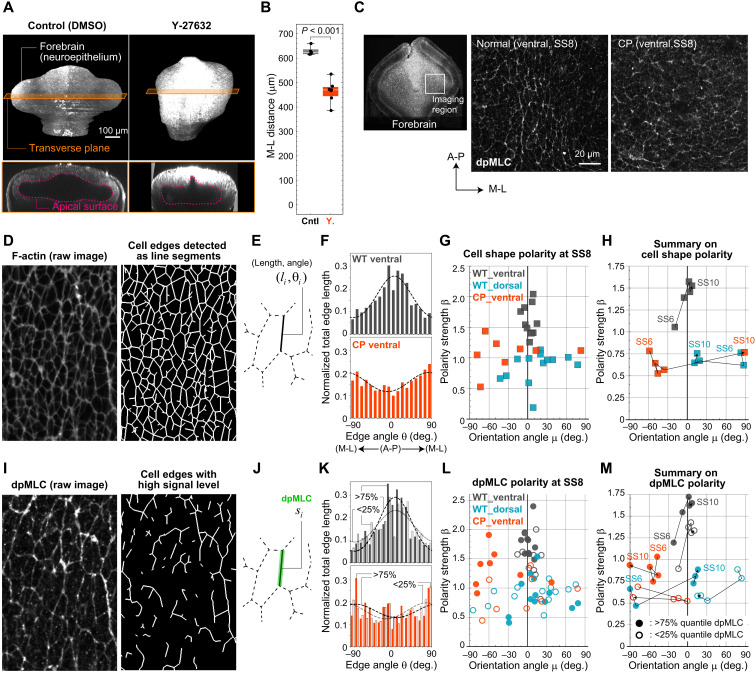
SHH-dependent cell polarity. (**A**) Comparison of forebrain morphology at SS10 for normal development and in the presence of Y-27632. (**B**) Quantification of M-L distance between left and right OV tips. (**C**) dpMLC immunostaining in the ventral neuroepithelium in the absence and presence of cyclopamine. From F-actin staining images, each cell edge was detected (**D**) and characterized by its length and angle (**E**). (**F**) For each sample, the sum of the lengths of edges facing in each direction was calculated and the result was fitted with a von Mises distribution with polarity strength β and orientation angle μ, which were used to quantify cell shape polarity. Top: Example of normal ventral tissue at SS8; bottom: ventral tissue at SS8 with cyclopamine treatment. WT, wild type. (**G**) Cell shape polarity at SS8 for each sample. (**H**) Summary of cell shape polarity. (**I** and **J**) Quantification of dpMLC level of each edge (J) and detection of edges with higher or lower signal levels (I) (see also fig. S6F). (**K**) Calculation of the polarity for two groups of edges having higher or lower signal levels. The top and bottom panels are for the untreated and treated samples, respectively, shown in (F). (**L**) Polarity for edge groups with higher or lower dpMLC levels for each sample at SS8. (**M**) Summary of dpMLC localization polarity. See text, Materials and Methods, and figs. S5 and S6 for details on image processing and analysis.

Recent mechanobiological studies reported the relevance of SHH signaling in regulating cell/tissue mechanics. For example, during zebrafish somitogenesis and eye disc formation in *Drosophila*, SHH regulates localization of myosin activity on apical surfaces of epithelial cells to induce apical constriction that results in large deformation of epithelial sheets (*12*–*14*). To find a link between SHH signaling and forebrain morphogenetic dynamics, we first examined whether myosin activity is needed for OV elongation and its relevance to SHH signaling. Inhibition of myosin phosphorylation by the ROCK inhibitor Y-27632 suppressed OV elongation [Fig. 2, A and B; inhibition of myosin II adenosine triphosphatase (ATPase) activity by blebbistatin is also shown in fig. S4], indicating that myosin activity is essential for OV elongation. We then examined the spatial distribution of di-phosphorylated myosin light chain (dpMLC), an indicator of activated myosin II (*15*, *16*), on the apical surface of neuroepithelium. Since we previously demonstrated that apical cell shape is elongated in the A-P direction (*11*), here we simultaneously monitored F-actin using phalloidin as a marker of cell membrane shape and dpMLC to quantify the polarity of cell shape and dpMLC localization (Fig. 2, C to M; figs. S5 and S6; and Materials and Methods).

From F-actin staining images, we detected cell edges as line segments (Fig. 2D) and determined the orientation and length of each edge (Fig. 2E). The sum of the lengths of edges facing in each direction was calculated, and the results were fitted with a von Mises distribution (Fig. 2F). The strength of polarity (β) and its orientation angle (μ) were calculated for each image (i.e., right or left OV region of each embryo) using the distribution parameters (Fig. 2G shows SS8 as an example). Furthermore, the mean value of dpMLC signals on each cell edge was calculated and defined as the signal intensity of that edge (Fig. 2J). Edges having higher dpMLC intensity (specifically, >75% quantile) and those with lower dpMLC intensity (<25% quantile) were selected (Fig. 2I, right panel shows edges having higher values), and, as in the F-actin case, the sum of lengths of edges facing in each direction was calculated, and the results were fitted with von Mises distributions to quantify the strength of polarity and its orientation angle (Fig. 2, K and L, for SS8). The different strength of polarity between these two groups (i.e., higher and lower edges) indicates that the polarized pattern of dpMLC reflects not only the polarity of cell shape but also the polarity of its subcellular localization.

From a structural perspective, during early development, the forebrain region is a nearly symmetrical tube dorsoventrally. However, in this period, expression of SHH, which is involved in dorsoventral (D-V) patterning of the NT, occurs only in the ventral part of the forebrain, and thus, its signaling activity is dorsoventrally asymmetric. In normal ventral tissues having higher SHH signaling activity (Fig. 1A) (*17*), cell shapes (or edges) show a clear polarity along the A-P axis, i.e., perpendicular to the axis of tissue deformation or OV elongation, especially at or after SS7, when OV elongation is remarkable (Fig. 2H and fig. S5, C and D). In addition, the edges with higher dpMLC intensity had higher A-P polarity than those with lower dpMLC intensity, indicating that subcellular dpMLC localization is also polarized (Fig. 2M and fig. S5, E and F). In contrast, normal dorsal tissues that have much lower SHH activity had substantially weaker polarity in cell shape and dpMLC localization, although the orientation angle is near the A-P axis at and after SS8 (Fig. 2, H and M, and fig. S5, C to F). In the presence of an SHH inhibitor, the clear A-P polarity of both cell shape and dpMLC seen for normal ventral tissue disappeared, and the orientation angle also deviated significantly from the A-P axis (Fig. 2, H and M, and fig. S5, C to F). Meanwhile, dpMLC itself remained localized to the cell edges (Fig. 2C). Together, these results indicate that SHH, which has D-V asymmetric expression, is involved in regulating cell polarity in the ventral neuroepithelium.

### D-V asymmetry in self-organization ability

**Fig. 3.**
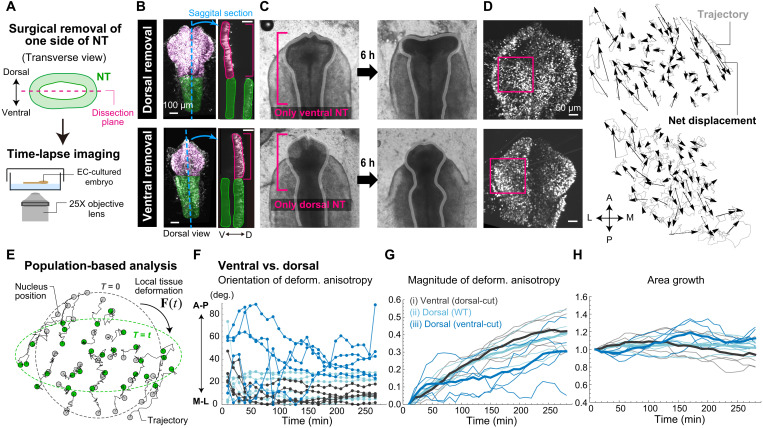
D-V asymmetric self-organization ability. (**A**) Schematic diagram of time-lapse imaging. (**B**) Dorsal and sagittal-sectional views of forebrain regions with the dorsal or ventral half removed. (**C**) Morphological changes within 6 hours of removal of the dorsal or ventral half. (**D**) Representative nuclei trajectories (Materials and Methods and fig. S8). (**E**) From the trajectories of several dozen cells, local tissue deformation was calculated. See Materials and Methods and fig. S8 for details. (**F** to **H**) Comparison of local deformation characteristics between ventral and dorsal forebrain tissues, (F) orientation of deformation anisotropy (angle from the M-L axis), (G) magnitude of deformation anisotropy, and (H) area growth on the plane perpendicular to the apico-basal axis, measured as the ratio to the area at the time of initial imaging.

We next addressed whether and how the SHH-dependent D-V asymmetry of cell polarity is related to tissue morphogenesis. First, we found that OV could elongate even when the distal tip and adjacent surface ectoderm were removed (fig. S7), indicating that OV elongation does not occur passively via an external pulling force from its end. Second, we observed that, even when the dorsal half of the forebrain was surgically removed, the ventral tissue still elongated in the M-L direction that is perpendicular to the A-P–oriented cell polarity. However, dorsal tissue alone that has reduced polarity did not show M-L elongation (Fig. 3, A to C). We quantified local tissue deformation around OV regions during these processes based on trajectory data for several tens of nuclei (Fig. 3D, fig. S8, and movies S1 and S2). Specifically, the local deformation was approximated as a linear transformation from the initial configuration (corresponding to the deformation gradient) that best describes the motion of the nuclei population, and the time evolution of deformation anisotropy and area growth was calculated over 4.5 hours from SS7 (Fig. 3, E to H; fig. S8; and Materials and Methods). The ventral tissue continued to elongate in the M-L direction and shrink in the A-P direction over the measurement period without the dorsal half (Fig. 3, F and G, black). In contrast, in the absence of ventral tissues, the dorsal tissues showed reduced deformation anisotropy, and the direction of deformation varied greatly along the time axis and between samples (Fig. 3, F and G, blue).

Meanwhile, in normal development, the dorsal tissues showed unidirectional elongation along the M-L axis and deformation anisotropy comparable to that seen for ventral tissues (Fig. 3, F and G, light blue). In all cases, apical area growth was minor (Fig. 3H). Together, these results show that ventral tissues that have higher SHH signaling activity can deform more actively along a specific direction, whereas dorsal tissues with much lower or almost no SHH signaling activity (*17*) have little active, unidirectional deformation. This D-V asymmetric self-organization ability indicates that ventral tissues are the driver of OV elongation in normal development.

### SHH regulates directional cell rearrangement

**Fig. 4.**
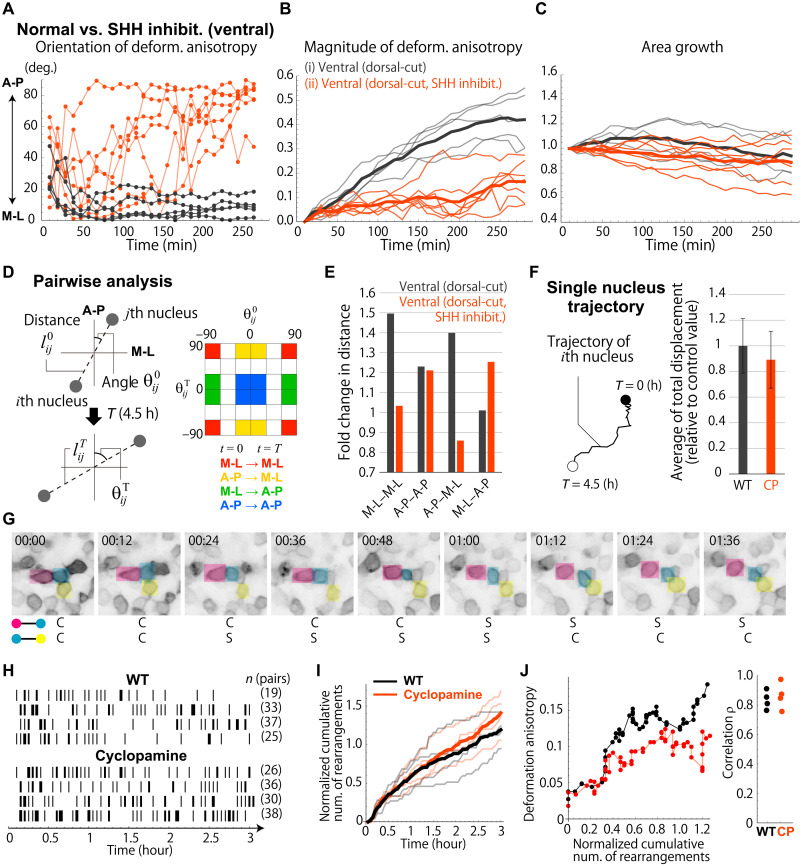
SHH-dependent directional cell rearrangement. (**A** to **C**) Comparison of local deformation characteristics of ventral tissues in the absence and presence of SHH inhibitor. (**D**) The extent of cell rearrangement was quantified from the distance and angular changes for each nucleus pair. (**E**) Dependence of fold change in inter-nuclei distance on the direction. Geometric SDs: 1.65 (M-L–M-L), 1.56 (M-L–A-P), 1.70 (A-P–M-L), and 1.77 (A-P–A-P) for normal ventral tissues and 1.55 (M-L–M-L), 2.01 (M-L–A-P), 1.72 (A-P–M-L), and 1.63 (A-P–A-P) for ventral tissues with cyclopamine treatment. (**F**) Cell motility was measured as the average of total displacement along the trajectory of individual nuclei. (**G**) Example of time-lapse imaging showing changes in cell adjacency relationships that occurred over time through cell rearrangement. C: in contact; S: separated (see Materials and Methods and fig. S9 for details). (**H**) Raster plots of changes in adjacency relationships within 3-min intervals. Bar thicknesses indicate the number of changes in each interval; one (thinnest) to four (thickest) times. (**I**) Time evolution of the cumulative number of cell rearrangements normalized by the number of cell pairs traced (black: normal; red: cyclopamine-treated). Thick lines show averages of four samples (represented by thin lines). (**J**) Correlation between cumulative number of rearrangements and deformation anisotropy (right) and an example (left).

To demonstrate that the self-organization ability of ventral tissues that drives OV elongation is dependent on SHH signaling, we then compared tissue and cellular dynamics in the presence/absence of SHH inhibition (Materials and Methods). Ventral tissue from embryos in which the dorsal half of the forebrain region was surgically removed was used for the analysis. We first did a similar analysis of local tissue deformation using trajectory data for nuclei populations included in the initial bulge of OV under SHH inhibition (Fig. 4, A to C, and movie S3). In the presence of SHH inhibition, substantial loss of tissue deformation unidirectionality and anisotropy with minor area growth was observed, which is consistent with the results for dorsal tissue alone that has a lower degree of SHH signaling (Fig. 3, F to H, blue). This result demonstrates that the self-organization ability of normal ventral tissue is regulated by SHH signaling.

As mentioned above, cell proliferation is not necessary for OV elongation. Moreover, we previously showed that changes in cell shape/size cannot explain the observed anisotropic tissue deformation pattern (fig. S3) (*11*). Thus, we next examined cell rearrangement patterns and cell motility in the absence/presence of SHH signaling based on data for nuclei and membranes. First, using nucleus trajectory data with much higher resolution, we calculated how the distance between each pair of nuclei that were located near each other (specifically within 25 μm) changes over time depending on the direction in which the pair is connected (Fig. 4D, left). Since changes in cell shape and size within the time period of interest were minor (fig. S8F), the clear direction dependence of the change in inter-nuclei distance means that cell rearrangement occurred in a specific direction. In particular, we focused on nucleus pairs that were oriented toward the M-L axis (−90° < θ < −60° or 60° < θ < 90°) or the A-P axis (−30° < θ < 30°) at the initial (*t* = 0, SS7) and final time (*t* = 4.5 hours) points and quantified the changes in their inter-nuclei distance (Fig. 4D, right). In normal ventral tissues, the distances between nucleus pairs oriented toward the M-L axis at the final time point were clearly increased, whereas under SHH inhibition those were essentially unchanged or slightly decreased (see M-L–M-L or A-P–M-L in Fig. 4E). Cell division and death during the measurement period was infrequent and likely had only a minor effect on local tissue deformation and cell rearrangement. These results indicate that, in normal ventral tissue, the tissue elongates in the M-L direction through SHH-dependent directional cell rearrangement. Furthermore, by measuring the motility of cells by total displacement along the trajectory of individual nuclei, we showed that the motility was comparable in the absence/presence of SHH inhibition (Fig. 4F and Materials and Methods). This result indicates that SHH inhibition suppresses OV elongation not by interfering with cell motility but rather by causing cells to lose polarity in their movement.

We next attempted to detect dynamic changes in adjacency relationships between cells by performing live imaging of embryos having cell membranes that were randomly labeled with myrVenus introduced by electroporation (Materials and Methods). Because of the deep-tissue imaging, the spatial resolution was not high enough to track all labeled cells, but we could manually trace trajectories of sufficient numbers of cells to quantify the number of changes in adjacency between cells by carefully observing multiple sectional images perpendicular to the apico-basal axis and considering that the neuroepithelium is a single-layered structure in which each cell maintains a columnar shape during the measurement period (Fig. 4G and fig. S9) (*11*). This frequency of the change in adjacency relationships can be a measure of cell motility. On the other hand, direct detection of unidirectional tissue elongation/shrinkage through cell rearrangements on a wider scale was difficult, although we could observe sparsely labeled cells moving apart (fig. S9D). Raster plots representing the occurrence of changes in adjacent relationships within every 3 min were produced, with the thickness of each vertical bar indicating the number of changes within each time interval (Fig. 4H). The time evolution of the cumulative number of cell rearrangements was also plotted (Fig. 4I). In both the absence and presence of SHH inhibitor, the cumulative number of rearrangements was proportional to time, showing that cell rearrangements occurred nearly constantly, and the rates with and without inhibitor were comparable (see graph slope in Fig. 4I). This result is consistent with the result that cell motility measured as total displacement along each nucleus trajectory is nearly independent of SHH signaling (Fig. 4F). Furthermore, in both the absence and presence of SHH inhibitor, the cumulative number of cell rearrangements was strongly correlated (*r* > 0.75) with the anisotropy of local tissue deformation calculated from trajectories of cells in the focal tissue patches (Fig. 4J). This result supports that tissue deformation is driven by cell rearrangement. Note that the amount of deformation was somewhat smaller than the results of calculations based on nucleus trajectory data (see Fig. 3G). This difference could be due to slower growth, as a stronger laser intensity (about 30-fold) was needed to detect fluorescent labeling of the cell membrane.

Together, in normal development, OV elongation along the M-L axis is driven by self-organization through active and directional cell rearrangement in ventral tissues. In embryos with SHH inhibition, loss of ventral self-organization ability due to randomization of the orientations of cell shape, dpMLC localization, and cellular movement prevents sufficient elongation and separation of the left and right OVs, consequently leading to fusion of the eyes around the midline. This conclusion about the importance of A-P–oriented cell polarity for OV elongation is also supported by the phenotype seen for treatment with calyculin A, an inhibitor of myosin light chain dephosphorylation that results in myosin activation (fig. S4). In the presence of calyculin A, the orientation angle of the polarity of both dpMLC and cell shape within the ventral tissue shifted by a few tens of degrees from the A-P axis to the M-L direction, and as a phenotype, OV elongation was suppressed, as was observed for treatment with the ROCK inhibitor Y-27632.

### SHH regulates cellular mechanosensation

**Fig. 5.**
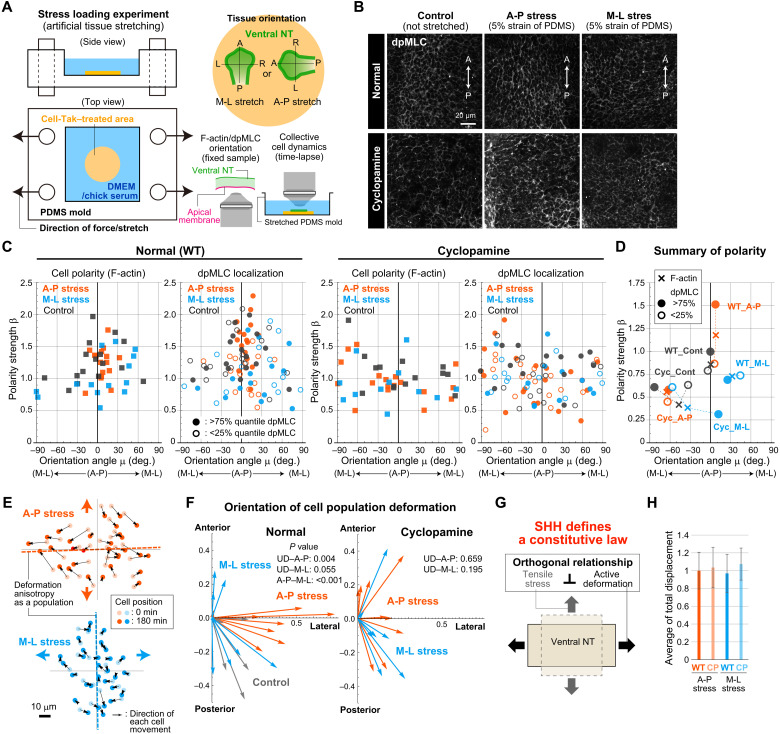
SHH-dependent stress responses. (**A**) Schematic diagram of the stress loading experiment (left) to examine responses to A-P or M-L stress (top right). To measure responses, the effects on cell polarity and collective cell motion were quantified for ventral OV regions in the absence and presence of cyclopamine (bottom right). (**B**) Immunostaining images of dpMLC for the control and samples under stress. (**C**) Quantification of polarity in cell shape and dpMLC localization for each sample in the absence and presence of cyclopamine treatment. (**D**) Summary of cell polarity. The orientation angle and polarity strength for the combined data from all samples for each case are shown. (**E**) Typical examples of cellular displacement during 3 hours under stress (see also fig. S10). (**F**) Orientation of cell population deformation. See Materials and Methods for statistical tests. UD, uniform distribution. (**G**) SHH defines a constitutive law that morphogenesis proceeds in a way that satisfies an orthogonal relationship between stress and active deformation through polarized cell motion. (**H**) Cell motility was measured as the average of total displacement along the trajectory of individual nuclei.

In SHH-inhibited embryos, anisotropic tissue deformation, which is essential for OV elongation, is impaired by loss of cell polarity that would occur in normal development. The essential question that remains unanswered is what factors determine the direction of the polarity. Recent mechanobiological studies suggest that tissue stress is involved in defining the direction of tissue polarity (*18*, *19*); in particular, phosphorylated myosin is known to orient in response to mechanical stimuli (*20*). Thus, we next examined cellular mechanical responses by artificially imposing stress on pieces of neuroepithelium cut from the ventral OV region that were pasted on a polydimethylsiloxane (PDMS) stretch chamber and then assessed whether/how SHH is involved in stress responses (Fig. 5A and Materials and Methods).

In normal tissues, cell shape and dpMLC localization had weak A-P polarization even in the absence of external stress (default state) (Fig. 5, B to D). Note that the polarity in dpMLC localization is judged on the basis of the difference in the polarity strength between edge groups with higher and lower dpMLC intensity. When stress was imposed in the A-P direction, the polarity of both cell shape and dpMLC was clearly enhanced (Fig. 5, B to D). Meanwhile, for M-L stress, the weak cell polarity along the A-P axis seen in the default state disappeared and the orientation angle for cell shape polarity shifted toward the M-L axis by around 30°. However, no clear polarity was seen for dpMLC, as evidenced by the equivalent polarity strengths seen for edges with higher and lower signal intensity (Fig. 5, B to D). These results demonstrate that, in normal tissues, neuroepithelial cells can sense stress direction, and the polarized patterns in cell shape and dpMLC localization can be markers for mechanosensation (*20*).

When A-P stress was imposed on the normal tissue patch, cells moved so that the tissue patch elongated in the M-L direction, i.e., perpendicular to the stress direction (Fig. 5, E and F; fig. S10; and movie S4). On the other hand, under M-L stress, local tissue deformation biased in the A-P direction was observed, but the specificity in the deformation orientation was smaller than that under A-P stress, as reflected in the *P* value when compared to the uniform distribution (*P* = 0.055) (Fig. 5, E and F; fig. S10; and movie S5). This result may be due to the lower cell polarity compared to that seen for A-P stress. These observations are counterintuitive since ordinary passive materials typically elongate along the axis parallel to the direction of tensile stress. The observed anisotropic tissue deformation orthogonal to the stress direction can only be achieved through active cellular movement. From the perspective of materials science, this orthogonal relationship between the directions of tensile tissue stress and deformation anisotropy (i.e., the tissue elongation direction) represents a constitutive law (i.e., the functional relationship between stress and strain), which is essential for early forebrain morphogenesis to proceed normally (Fig. 5G). Cell motility, measured in terms of the total displacement along the trajectory of individual nuclei, was comparable between A-P and M-L stresses (Fig. 5H).

In the presence of SHH inhibitor, no clear polarity in cell shape and dpMLC localization was observed regardless of the direction of external stress (Fig. 5, C and D). The direction of collective cell motion (or local tissue deformation) was also random and varied among samples (Fig. 5F, fig. S10, and movies S6 and S7), although cell movement itself was observed (Fig. 5H and fig. S10). Together, the results of the stress loading test revealed previously unidentified roles for SHH signaling in conferring on neuroepithelial cells’ mechanosensation activity and in defining a constitutive law for early forebrain morphogenesis. These findings consistently explain the random orientation of cell shape, dpMLC localization, cell motion, and the resultant failure of OV elongation observed in the SHH-inhibited embryos.

### Stress patterns in 3D neuroepithelium

**Fig. 6.**
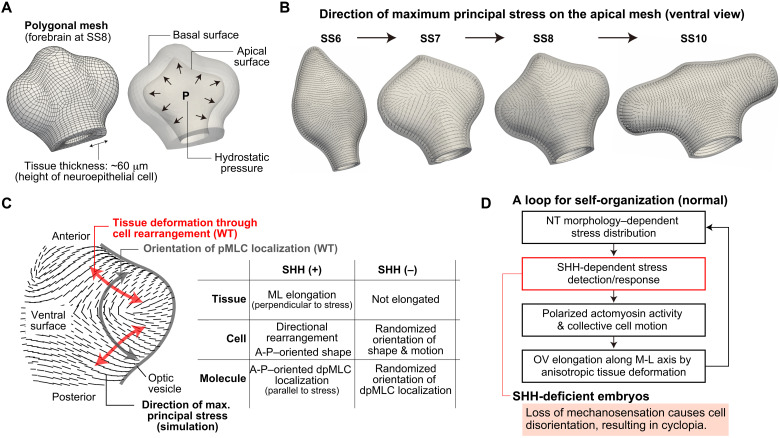
3D stress patterns predicted by mechanical simulations and summary of a previously unidentified role for SHH in tissue/cell dynamics. (**A**) Example of a 3D polygonal mesh (left). Tissue stress under weak hydrostatic pressure was calculated (right; see also fig. S11). (**B**) Spatial patterns of the direction of maximum principal stress on the apical surface obtained by simulations. (**C**) Summary of the effects of deficient SHH signaling on behaviors at different spatial scales. (**D**) Loop for the self-organization process during normal forebrain development. In SHH-deficient embryos, loss of mechanosensation causes cell disorientation, resulting in cyclopia.

Last, we examined stress patterns in the 3D neuroepithelium. Since, as in many organs, direct stress measurement of deep tissues was difficult (*21*, *22*), we estimated the stress orientation pattern using mechanical simulations (Materials and Methods). Hyperelasticity was adopted as a physical property of the neuroepithelium, in accordance with its use in studies of the morphogenesis of other organs such as gut looping, brain gyrus formation, and heart tube C-looping (*23*–*25*). Since the NT is filled with biofluid, tissue stress under weak hydrostatic pressure was calculated using a finite element method (Fig. 6A and fig. S11). In examining the spatial pattern of the maximum principal stress direction for the apical side during normal OV elongation (Fig. 6B and fig. S12), the orientation patterns reflect the 3D neuroepithelial morphology well; in particular, at SS8 and SS10, when the OV bulges are more distinct, the A-P–oriented stress pattern and the orthogonal relationship between the directions of stress and tissue elongation were clearer around the OV regions. This stress orientation pattern remained almost unchanged even when the magnitude of hydrostatic pressure was changed, showing the robustness of the pattern (fig. S12).

Together, we concluded that (i) in normal development, neuroepithelial cells can sense tissue stress in an SHH-dependent manner, and OVs elongate through cell rearrangement orthogonal to the orientations of cell shape and dpMLC localization that reflects tissue stress pattern, and (ii) in SHH-deficient embryos, cells cannot correctly detect the stress direction, which is reflected by the randomly oriented patterns of cell shape and dpMLC localization (Fig. 6, C and D). This inability to detect the direction of stress results in random cell motion and failure of tissue elongation in a specific direction, consequently leading to a cyclopia-like phenotype due to insufficient separation of the left and right OV tips.

## DISCUSSION

During organ development, appropriate mutual feedback among tissue/cell geometry, mechanics, and biochemistry drives morphogenesis in a self-organized fashion (*26*). In the context of early forebrain morphogenesis, here, we examined the role of SHH, particularly in terms of physical tissue/cell dynamics. We discovered a previously unknown function for SHH, which can confer mechanosensation ability on cells and thereby control polarized cell motion. This role defines a morphogenetic law, i.e., an orthogonal relationship between the directions of tissue stress and active deformation. Inhibition of SHH leads to cell disorientation and ultimately to a cyclopia phenotype.

Mechanosensation involves sensing of stress anisotropy, transduction of the sensed information, and cellular responses depending on the information (*27*, *28*). Elucidating the detailed molecular mechanism of SHH-dependent mechanosensation is an important future challenge. SHH signaling was previously shown to be involved in cell morphological changes and cell motility via Rho pathway–dependent myosin phosphorylation (*12*, *29*). Furthermore, mechanical stimulation was reported to be involved in Rho pathway–mediated myosin phosphorylation (*20*). These findings suggest that investigating how Rho pathway activation and polarization of phosphorylated myosin localization depends on SHH signaling and mechanical stimulus may provide clues to elucidate the molecular mechanisms linking SHH signaling and mechanosensation. Once formed in a tissue, polarity could induce directional cell rearrangement, resulting in anisotropic tissue elongation. In *Drosophila* germ-band extension and chick NT closure, polarized phosphorylated myosin patterns have been reported to cause active tissue deformation (*16*, *30*). This issue may also be related to efficient organoid production; for instance, in the study of retinal organoid engineering, the relevance of SHH signaling to self-organization ability was noted (*31*, *32*).

Similar mechanisms may also function in normal development and congenital malformations of other organs/species since anisotropic tissue deformation plays a central role in the morphogenesis of various vertebrate organs such as limbs, the lower jaw, heart tube, and teeth, where SHH is a key signaling molecule (*25*, *33*–*35*). In addition, typical morphogens other than SHH have been reported to be involved in regulating mechanical responses. For instance, Wnt was reported to modulate sensitivity to shear stress (*36*) and to reinforce mechanocoupling between adherens junctions and the actin cytoskeleton (*37*) during vascular morphogenesis/remodeling. Fibroblast growth factor (FGF) was shown to regulate collective cell dynamics through cytoskeletal remodeling (*38*–*40*). These reports and our findings emphasize roles of morphogens in defining tissue/cell physics and morphogenetic laws beyond the traditional biochemical roles in the regulation of differentiation and spatial patterning.

## MATERIALS AND METHODS

### Embryo preparation and culture methods

Fertilized chicken eggs from Shiroyama Farm and Inoue Egg Farm were incubated in a humidified incubator at 38°C to obtain Hamburger and Hamilton (HH) stage 4 embryos (*41*). For subsequent cell labeling and 3D imaging, the embryos were explanted using a modified Early Chick (EC) culture method (*42*) and grown to the desired stage for all experiments, except for quantitative polymerase chain reaction (qPCR) (Fig. 1E) and 3D imaging (Fig. 1D) for which HH16 was used. Since it was difficult for embryos to develop normally to later stages (including HH16) with the EC culture method, a modified protocol for the Cornish pasty (MC) culture method (*43*) was used instead. The experiments were approved by the Ethics Committee of RIKEN Center for Biosystems Dynamics Research and performed under the institutional ethical guidelines.

### Construction of vectors for fluorescent protein expression and cell labeling

To label neuroepithelial cells within the forebrain region, we used two kinds of plasmids containing the fluorescent proteins and sox2-enhancer (N2 enhancer): pN2-myrVenus described in our previous study (*11*) was used for membrane labeling, and pN2-H2BVenus, prepared as follows, was used for nuclei labeling. We replaced the myrVenus gene in the pN2-myrVenus construct with the H2BVenus gene amplified by PCR to yield pN2-H2BVenus (see table S1 for PCR primers). The plasmids were electroporated into the prospective forebrain region using a CUY21EX electroporator (BEX) with the following pulse sequence: a single 50-ms poration pulse with 7-V amplitude and five 50-ms driving pulses with 3-V amplitude separated by 50-ms intervals.

### Inhibition of SHH signaling

We inhibited SHH signaling in three ways: addition of the *smoothened* antagonists (i) cyclopamine (LKT Laboratories, C9710) or (ii) sonidegib (Abcam, ab269876), or (iii) electroporation of small interfering RNA (siRNA) against *smoothened* (siRNA-*SMO*). Results for cyclopamine are discussed in the main text and figures, and similar results, specifically suppression of OV elongation and disappearance in polarity in cell shape and phosphorylated myosin localization, were obtained with sonidegib or siRNA-*SMO* (fig. S2). Cyclopamine (100 μM in 1% EtOH) or sonidegib (100 μM in 1% dimethyl sulfoxide) was added to embryos at HH4 or HH10 (=SS10), and the embryos were then incubated until the desired stages. The embryos were incubated with 2.5 μM cyclopamine in MC culture medium until HH16 for experiments displayed in Fig. 1 (D and E). The following siRNA sequence against *smoothened* was used: GCGUCAUCAUCUUUGUCAUUGUCUA (sense) and UAGACAAUGACAAAGAUGAUGACGC (antisense). The siRNA (4 nM) was electroporated with the pCAGGS-H2BEGFP vector (*25*), which encodes green fluorescent protein (GFP) as a tracer, into the forebrain region. A negative control siRNA (Thermo Fisher Scientific, 12935300) was electroporated into control embryos. The conditions for electroporation are the same as those used for the fluorescent protein described above.

### Inhibition/activation of myosin activity

The following chemical compounds were used to manipulate actomyosin activity: (i) Y-27632 (Wako, 257-00513) to inhibit myosin light chain phosphorylation, (ii) calyculin A (Wako, 038-14453) to inhibit myosin light chain dephosphorylation (i.e., activate myosin activity), and (iii) blebbistatin (Sigma-Aldrich, B0560) to suppress myosin contractility. Y-27632 (100 μM), calyculin A (100 nM), or blebbistatin (100 μM) was added to the culture system at SS6. Results for Y-27632 treatment are shown in the main text and figures. Blebbistatin treatment similarly inhibited OV elongation, and the phenotypes were similar; OV was somewhat widened in the A-P direction in addition to the suppressed elongation in the M-L direction (fig. S4). In ventral tissues of embryos treated with calyculin A, the orientation angle of both phosphorylated myosin localization and cell shape polarity shifted a few tens of degrees from the A-P axis to the M-L direction, and, as a phonotype, OV elongation was suppressed similarly to that seen for Y-27632 or blebbistatin (fig. S4). These results support that polarity in phosphorylated myosin localization and cell shape, as well as myosin contractility are essential for OV elongation.

### Light-sheet imaging

To examine the effect of SHH inhibition on the phenotype at HH16, embryos were observed by light sheet microscopy with a Zeiss Lightsheet Z.1 (ZEISS) microscope (Fig. 1D). Because HH16 embryos are large, the CUBIC method (*44*) was used to render them transparent to allow deep-tissue imaging. Embryos were fixed with 4% paraformaldehyde (PFA) (Wako, 162-16065) in phosphate-buffered saline (PBS) (Nippon Gene, 314-90185) for 1 hour and washed twice with PBS before treatment with CUBIC-I solution for 24 hours at 37°C. Then, the embryos were stained with NucGreen Dead 488 ReadyProbes Reagent (Thermo Fisher Scientific, R37109) in CUBIC-I solution for 24 hours at 37°C and washed twice with PBS. For imaging, the embryos were mounted with 0.8% SeaPlaque GTG agarose (Lonza, 50111)/PBS.

### RNA isolation and RT-qPCR

Total RNA was extracted from dissected forebrain regions (dorsal or ventral tissue) using a PureLink RNA mini kit (Thermo Fisher Scientific, 12183018A) following the manufacturer’s instructions. cDNA was synthesized using a PrimeScript RT reagent kit (Takara, RR037A) from 500 ng of total RNA. Reverse transcription qPCR (RT-qPCR) was performed using Power SYBR Green Master Mix (Thermo Fisher Scientific, 4367659) and the 7500 Fast Real-Time PCR System (Applied Biosystems; see table S2 for RT-qPCR primers).

### Physical ablation assay

To investigate the self-organization ability of forebrain neuroepithelium during stages in which OV formation/elongation (i.e., SS7 to SS10) occurs, at around SS7, we used microscissors (Fine Science Tools) to surgically remove the dorsal half (Figs. 3, F to H, and 4, A to C), ventral half (Fig. 3, F to H), or the distal tip of the OV (fig. S7) and observed how this removal affected morphogenesis and cell motion.

### Live imaging by multiphoton microscopy

Live 3D imaging was performed using an upright microscope (FV1000MPE, Olympus) or an inverted microscope equipped with an Olympus 25×/numerical aperture 1.05 XLPLN25XWMP objective and a multiphoton femtosecond laser (excitation wavelength, 920 nm; Mai Tai DeepSee eHP, Spectra-Physics). An electroporated embryo with pN2-myrVenus was immersed in 1× PBS and live-imaged using an upright microscope (Fig. 1B). The 3D morphology of the NT was determined by stacking 80 optical section images along the *Z* axis at 5-μm intervals. For each *Z* level, two *XY* images (512 × 512 pixels each) were tiled to include the entire region of the prospective brain. All images were taken at room temperature. Nuclei trajectories (Fig. 3D and fig. S8, A to D) and temporal changes in cell-cell adjacency based on membrane information (Fig. 4G and fig. S9, B and D) were live-imaged using an inverted microscope equipped with a stage-top incubator (TOKAI HIT). In both cases, an electroporated embryo with pN2-H2BVenus or pN2-myrVenus was placed dorsal side down on EC culture medium prepared on glass-bottom dishes (IWAKI, 3971-035). 3D nuclei trajectories were imaged by stacking 120 to 160 optical section images along the *Z* axis at 2.5-μm intervals. For each *Z* level, four *XY* images (320 × 320 pixels each) were tiled to include the entire region of the prospective forebrain. Images were acquired at 10-min intervals (Fig. 3D and fig. S8). The 3D cell membrane dynamics of 80 to 100 sections were imaged (~3-μm intervals) along the *Z* axis, and the information was used for determining whether two cells are in contact (Fig. 4G and fig. S9). For each *Z* level, an *XY* image (1024 × 1024 pixels each) was acquired every 3 min. 3D nuclei trajectories under an external stress were imaged using an upright microscope equipped with a stage-top incubator (TOKAI HIT) with stacking of optical section images along the *Z* axis at 2.5-μm intervals and a total of 120 to 160 sections (Fig. 5E and fig. S10). For each *Z* level, a 512 × 512 pixel *XY* image was acquired.

### Immunofluorescence assay and antibodies

To investigate the orientation of dpMLC localization and cell shape, immunostaining was performed according to a previously reported method (*16*) with the following modifications. Embryos were fixed with 4% PFA in PBS for 1 hour and washed twice with PBS. The dorsal and ventral halves of the forebrain were dissected, and each half was permeabilized with 0.5% Triton X-100 (Wako, 162-24755) in PBS for 30 min at room temperature. The halves were then washed with TBST [0.1% Tween 20 (Wako, 166-21213) in tris-buffered saline (Takara, T903)] before blocking in 10% normal donkey serum (Merck, D9663-10ML)/3% bovine serum albumin (BSA) (Merck, A9647) in TBST for 1 hour at room temperature and staining with primary antibodies overnight at 4°C. Primary antibodies were diluted in Can Get Signal (TOYOBO, NKB-601). Samples were then washed with TBST three times and stained with secondary antibodies for 1 hour at room temperature. Secondary antibodies and Alexa Fluor 568 phalloidin (Thermo Fisher Scientific, 12380) were diluted in 5% normal donkey serum/1.5% BSA in TBST, washed with TBST three times, and stained with 4′,6-diamidino-2-phenylindole (DAPI) (Immunobioscience, AR-6501-02) for nuclear counterstaining for 5 min at room temperature. All images were taken using an OLYMPUS FV3000 instrument. The following primary and secondary antibodies were used: anti-dpMLC antibody (Cell Signaling Technology, 3674S) and donkey anti-rabbit immunoglobulin G Alexa Fluor 647 (Thermo Fisher Scientific, A-31573).

### Quantification of cell polarity

Quantification of the polarity of dpMLC localization and cell shape was performed as follows. First, from phalloidin (F-actin) staining images, cell edges were detected as line segment (fig. S6A). Specifically, using Fiji software, the raw images were binarized and eroded. Then, after filling small holes, the images were skeletonized to detect cell edges. Since the detected edges could be winding, they were approximated as line segments by linearly connecting both ends of each cell edge, which enabled characterization of each edge by its orientation and length (fig. S6B). For each direction (precisely for each bin of direction), the sum of the lengths of all the edges facing in that direction was calculated and the result was fitted with a von Mises distribution (fig. S6C). The strength of polarity (β) and its orientation angle (μ) were calculated for each image (i.e., right or left OV region of each embryo) using the distribution parameters (fig. S6C; see also fig. S6, D and E, for the distribution shapes with different values of β and some F-actin patterns with different values of β and μ, respectively). For each cell edge, the mean value of the dpMLC signal on it was calculated and defined as the signal intensity of that edge (fig. S6, F and G). Note that each line segment representing a cell edge was dilated by one pixel in the calculation of dpMLC level on the segment. Edges with higher (>75% quantile) and lower (<25% quantile) dpMLC intensity were selected (fig. S6F), and, as for F-actin, for each direction, the sum of edges facing that direction was calculated, and the results were fitted with von Mises distributions to quantify the strength of polarity and the orientation angle (fig. S6H). The different polarity strengths for these two groups (i.e., higher and lower edges) mean that the polarized pattern in dpMLC not only reflects the cell shape polarity but also is derived from the subcellular localization polarity. In Figs. 2 (G and L) and 5C and figs. S2 (D and H), S4D, and S5 (C and E), the values of β and μ are plotted for each sample. In Figs. 2 (H and M) and 5D and fig. S5 (D and F) (i.e., graphs for summary), each point represents the polarity strength and orientation angle for the combined data from all samples for each case.

### Cell tracking, calculation of local tissue deformation, and evaluation of cell rearrangement and cell motility

Nuclei tracking was performed using Imaris 7.6 (Bitplane; Figs. 3D and 5E and figs. S8, A to D, and S10, B to E). We manually tracked dozens of nuclei within a focal patch, and the tissue drift was removed by subtracting the global average of the cell movement. After the tissue drift was removed, the local tissue deformation was calculated from the cell trajectory data. Here, ***x****_i_*(*t*) = (*x_i_*(*t*), *y_i_*(*t*))*^T^* represents the position of *i*th cell (*i* = 1,…, *n*) at time *t*, where *T* represents the transpose of the vector. The local tissue deformation (or deformation gradient tensor) **F**(*t*) with the initial position as a reference was assumed to be given by ***x****_i_*(*t*) = **F**(*t*)***X****_i_* + ξ, where ***X****_i_* = (*X_i_*, *Y_i_*)*^T^* ≡ ***x****_i_*(0) is the initial position of each cell and ξ is noise following a multivariate Gaussian distribution that is uncorrelated in time and space. The deformation gradient that minimizes the sum of squared errors ∑*_i_*‖***x****_i_*(*t*) − **F**(*t*)***X****_i_*‖^2^ is

$$\hat{F}(t)=(\begin{array}{c} x_{1}(t)x_{2}(t)\cdots x_{n}(t)\\ y_{1}(t)y_{2}(t)\cdots y_{n}(t) \end{array}){\left(\begin{array}{c} X_{1}X_{2}\cdots X_{n}\\ Y_{1}Y_{2}\cdots Y_{n} \end{array}\right)}^{+}$$

where the “+” symbol represents the generalized inverse matrix. The area growth and deformation anisotropy were calculated as quantities characterizing local tissue deformation. The former was defined by det**F**. The magnitude of the latter was defined as $1-\sqrt{\lambda_{2}/\lambda_{1}}$ using the square root of the eigenvalues λ_1_ and λ_2_ (λ_1_ ≥ λ_2_) of the right Cauchy-Green deformation tensor **C** ≡ **F***^T^***F**, and the direction was defined by that of the eigenvector, **ν**_1_, corresponding to λ_1_ (see also fig. S8E).

To evaluate cell rearrangement patterns using nucleus trajectory data, we calculated how the distance between each pair of nuclei within 25 μm of each other changes in time depending on the direction in which the pair is connected (Fig. 4, D and E). Since cell shape/size changes within the measurement time of interest are minor (fig. S8F), a clear directional dependence of the change in inter-nuclei distance means that cell rearrangement occurs in a specific direction. We particularly focused on nuclei pairs having initial (*t* = 0, SS7) and final time (*t* = 4.5 hours) orientations toward the M-L axis (−90° < θ < −60° or 60° < θ < 90°) or the A-P axis (−30° < θ < 30°) and quantified their distance changes (Fig. 4D). The geometric mean of the ratio of the inter-nuclei distance at the initial and final time points was calculated for each case (Fig. 4E). The geometric SDs are described in the figure legends. We then evaluated cell motility by assessing total displacement along the trajectory of individual nuclei. Results for calculation of total displacement may be affected by errors in the recognition of nuclei positions. Therefore, we also performed the calculation for the cases in which, for each nucleus, noise obeying 2D isotropic normal distribution with 0.5- or 1.0-μm SD was added to its positional coordinate data at each time point as a recognition error during image processing. We confirmed that the impact of these recognition errors on the value for cell motility relative to the case for normal ventral tissues (Figs. 4F and 5H) is minor.

To quantify changes in cell-cell adjacency relationships, we tracked cells based on the information of cell membranes randomly labeled with myrVenus introduced by electroporation using the TrackMate plugin (*45*) in Fiji software (Fig. 4G and fig. S9). As mentioned in the main text, the spatial resolution of microscopic images was not high compared to the nuclei data due to the deep-tissue imaging, but we could count the number of changes in the adjacency between cells by carefully observing multiple sectional images perpendicular to the apico-basal axis. A pair of cells that are in contact can be separated by the intercalation of another cell, but then either regain contact or remain separated. Here, we evaluated cell motility by the frequency with which each cell pair changed between contact and noncontact states. In Fig. 4H, for each sample from one embryo, the adjacency of dozens of cell pairs was observed. In the raster plot, the thickness of each vertical bar shows the number of pairs that changed contact status since the previous time point (i.e., 3 min before).

### Measurement of stress response in stress loading tests

Cellular stress responses were investigated by artificially applying external force to ventral forebrain tissues. Ventral forebrain tissues at SS3/SS4, which is the stage before NT closure around the forebrain region is completed, were isolated. Each of these tissues was attached to a PDMS membrane (STREX, STB-CH-04-H5) on a cell stretching device (STREX, STB-100) by coating the membrane with Cell-Tak (Corning, 354241). The tissues remained attached to the device during culture for 30 min in Dulbecco’s modified Eagle’s medium (DMEM) (Nacalai, 08489-45)/1% chick serum (Thermo Fisher Scientific, 16110082) at 37°C in a CO_2_ incubator. Then, we applied stress to the tissues to induce a 5% strain along the A-P or M-L axis of the forebrain, and the tissues were cultured again in the incubator under the same stress until a time corresponding to SS7 and SS8 in normal development was reached. To investigate the polarity in dpMLC localization and cell shape (Fig. 5, B to D), the tissues were fixed with 4% PFA/PBS 5.5 hours after the onset of stress loading. Subsequent analysis to quantify the polarity was carried out as described above. For imaging and quantification of collective cell motion (Fig. 5, E and F, and fig. S10, B to E), nuclei were labeled by electroporating plasmids (pN2-H2BVenus) into the tissues before attachment to the PDMS membrane. Time-lapse imaging was started 4 hours after stress loading onset, and images were collected every 5 min for 3 hours. The tissues were maintained under stress during the time-lapse imaging. The imaging conditions described in the “Live imaging by multiphoton microscopy” section were used. In the default state (i.e., the control condition without stress loading), the strength of cell polarity is smaller than that for in vivo (Fig. 2, H and M, and fig. S5, D and F) likely due to differences in experimental conditions and tissue processing between in vivo and stress loading tests.

### Mechanical simulation to calculate stress distribution within the forebrain region

Since direct stress measurement (e.g., using oil microdroplets or by laser ablation) of deep tissues was difficult, we estimated the stress orientation pattern using mechanical simulations. To calculate stress distribution within the forebrain region, we modeled this region as a thick-walled (about 60 mm), slightly compressible hyperelastic (Neo-Hookean) material with the strain energy function Ψ defined as (fig. S11)

$$\Psi (J,{\bar{I}}_{1})=\frac{\kappa }{2}{\left(J-1\right)}^{2}+c({\bar{I}}_{1}-3)$$

J=detF

$${\bar{I}}_{1}=\text{tr}({\bar{F}}^{T}\bar{F})$$

$$\bar{F}=J^{-1/3}F$$

where **F** is the deformation gradient tensor and κ = 1.0 × 10^4^ [kPa] and *c* = 1.0 × 10^2^ [kPa] are material constants (with biologically plausible values chosen). The 3D polygonal models were obtained on the basis of two-photon microscopy images of chick forebrain regions at SS6 to SS8 and SS10. Using a finite element method, we numerically solved the mechanical balance under a small hydrostatic pressure *p* within the forebrain cavity (i.e., load boundary condition). We tested 10 different values of *p* from 1 [kPa] to 10 [kPa] and confirmed that the spatial patterns of tissue stress (precisely the directions of principal stresses) were similar regardless of the value of *p*, showing the robustness of stress patterns that reflect the 3D neuroepithelial morphologies (fig. S12). In Fig. 6B, the results for *p* = 1 [kPa] are shown. Polygon models comprising three layers of hexahedral elements were used for our mechanical simulation (fig. S11). We adopted the displacement boundary condition that the posterior ends can move only in the transverse plane.

### Sample sizes and statistical tests

The sample sizes for SS6, SS7, SS8, SS9, and SS10 are eight, five, five, six, and five, respectively, for normal development and seven for all stages in cyclopamine-treated embryos (Fig. 1B). The sample sizes for conditions (i), (ii), and (iii) are seven, eight, and seven, respectively, for the quantification of phenotype (δ_L_), and those for the qPCR assay are eight for each condition and each gene (Fig. 1E). In Fig. 3 (F to H), the sample sizes are five, six, and five for conditions (i), (ii), and (iii), respectively, and in Fig. 4 (A to C), the sample size is seven for the cyclopamine-treated condition. In Fig. 5C, the sample sizes for A-P stress, M-L stress, and control are 16, 14, and 16, respectively, for tissues from normal embryos, and 18, 12, and 14, respectively, for tissues from cyclopamine-treated embryos. In Fig. 5F, the sample sizes for A-P stress, M-L stress, and control are eight, eight, and five, respectively, for tissues from normal embryos. For cyclopamine-treated embryos, we compared the difference in the response to A-P and M-L stresses, for which the sample sizes were nine and seven, respectively. We carried out two types of statistical tests: (i) UD–A-P/M-L in which the result for each case (A-P stress or M-L stress) was statistically tested under the null hypothesis that the data were sampled from the uniform distribution (UD), and *P* values were calculated using 10,000 numerically generated datasets in which the angle was sampled from the uniform distribution and the anisotropy was resampled from the anisotropy data for each case, and (ii) A-P–M-L in which results for A-P stress and M-L stress were compared under the null hypothesis that the data for A-P obey the same distribution as that seen for M-L. *P* values were numerically calculated using 1000 bootstrap sample sets resampled from the M-L data.

## Notes

View/request a protocol for this paper from *Bio-protocol*.
