# Supplementary material for: Cell disorientation by loss of SHH-dependent mechanosensation causes cyclopia
Source: Sci Adv. 2022 Jul 13;8(28):eabn2330. doi: 10.1126/sciadv.abn2330 (PMC9278851; doi:10.1126/sciadv.abn2330)
Supplement: Supplementary file 1 — Figs. S1 to S12 Tables S1 and S2 [file sciadv.abn2330_sm.pdf]

Science Advances
AAAS

Supplementary Materials for

Cell disorientation by loss of SHH-dependent mechanosensation causes cyclopia

Daisuke Ohtsuka *et al.*

Corresponding author: Yoshihiro Morishita, yoshihiro.morishita@riken.jp;
Daisuke Ohtsuka, daisuke.ohtsuka@riken.jp

Sci. Adv. **8**, eabn2330 (2022)
DOI: 10.1126/sciadv.abn2330

The PDF file includes:

Legends for movies S1 to S7
Figs. S1 to S12
Tables S1 and S2

Other Supplementary Material for this manuscript includes the following:

Movies S1 to S7

Legends for Figs. S1 to S12

Fig. S1. Morphological changes in the forebrain region during optic vesicle (OV) formation/elongation phase for normal development and following cyclopamine treatment (an inhibitor of the SHH signaling pathway).
A, B: Anterior of embryo (top), extracted forebrain region (middle), and transverse-sectional view of forebrain (bottom) from somite stage (SS) 6 to SS10 during normal development (**A**) and with cyclopamine treatment (**B**). Chick embryo at SS6 (**A**, right). **C**: Distance between the centers of the left and right lenses. (i)-(iii) are the same as Fig. 1E, and (iv) shows the case in which cyclopamine was added at SS10 (=HH10), and not before. *: $p<0.001$; **: $p=0.004$. Importantly, in case (iv), no fusion of the left and right lenses was observed. **D**: Morphological change in apical surface of the forebrain region during normal development. Surface representations were obtained by tracing two-photon microscopy images followed by smoothing using spherical harmonics expansion.

Fig. S2. Phenotypes with inhibition of SHH signaling by sonidegib or siRNA against *smoothened* (siRNA-*SMO*).
A-**D**. Results for sonidegib treatment; examples represent the effects on morphology (**A**) and dpMLC localization pattern (**C**), and quantification of ML-distance (**B**) and polarity in cell shape and dpMLC localization (**D**). $\bar{\mu}$ and $\bar{\beta}$ show the orientation angle and polarity strength, respectively, for the combined data from all samples for each case. See also Fig. S6 for image processing. **E**-**H**. Results for siRNA-*SMO* treatment; examples represent the effects on morphology (**E**) and dpMLC localization pattern (**G**), and quantification of ML-distance (**F**) and polarity in cell shape and dpMLC localization (**H**).

Fig. S3. A brief summary of our previous work.
All figures in Figure S3 are from our previous paper published in (11) and used in accordance with CC BY 4.0. The rose diagrams in **C** present all data including those for dorsal and ventral cells. As revealed in this study, cell shape polarity has D-V asymmetry (see Fig. 2, **G** and **H**).

Fig. S4. Effect of inhibition/activation of myosin activity.
A: Comparison of forebrain morphology at SS10 for normal development and in the presence of Y-27632, blebbistatin or calyculin A. All agents were added at SS6. Figures for the control and Y-27632 are the same as Fig. 2**A**. **B**: Quantification of medio-lateral distance between left and right OV tips. The data for the control and Y27632 are the same as Fig. 2**B**. **C**, **D**: Typical dpMLC localization patterns (**C**) and quantification of polarity in cell shape and dpMLC localization at SS8 (**D**) for control and calyculin A treatment. $\bar{\mu}$ and $\bar{\beta}$ show the orientation angle and polarity strength, respectively, for the combined data from all samples for each case. See also Fig. S6 for image processing. It should be noted that when Y-26732 was added, the phosphorylation level was much lower than that for the control, and that blebbistatin does not act on phosphorylation itself.

Fig. S5. Quantification of cell polarity.
A: Schematic diagram of experimental procedure (modified from Fig. 3**A**). **B**: Examples of dpMLC localization patterns on the ventral/dorsal apical surface for normal development and with cyclopamine treatment. **C**, **E**: Quantification of polarity in cell shape (**C**) and dpMLC

localization (**E**) for each sample; the left and right tissues were counted as separate samples for each embryo. See Fig. S6 for image processing. **D**, **F**: Summary of cell shape polarity (**D**) and dpMLC polarity (**F**); The orientation angle and polarity strength for the combined data from all samples for each case are shown.

Fig. S6. Image processing for the quantification of polarity in cell shape and dpMLC localization.
A: Procedures for detecting cell edges as linear segments from F-actin images (see Materials and Methods for details). **B**: Each cell edge is characterized by its length and angle. **C**: For each direction (more precisely, for each bin of direction), the sum of the lengths of the edges facing that direction (i.e., included in each bin) was calculated and the result was fitted with a von Mises distribution, which is characterized by polarity strength β and orientation angle μ. **D**: Dependence of distribution shape on β. **E**: Examples of F-actin images with different values of β and μ shown for visual understanding. **F**, **G**: For each cell edge, the mean value of the dpMLC signal on it was calculated and defined as the signal intensity of that edge (**G**). Edges with higher (> 75%-quantile) and lower (< 25%-quantile) dpMLC intensity were selected (**F**). The left and middle images shown in (**F**) is the same as Fig. 2**I**. **H**: As for F-actin, for each direction, the sum of edges facing in that direction was calculated, and the results were fitted with von Mises distributions to quantify the strength of polarity and its orientation angle. **I**: An example of image processing using data for a sample at SS8 with cyclopamine treatment that corresponds to the graphs shown in Fig. 2**F** and 2**K**.

Fig. S7. OV elongates even when the distal tip and adjacent surface ectoderm are cut off.
A: Areas to be surgically removed are outlined in red. **B**: An example of an embryo in which the tip of the right OV and adjacent surface ectoderm were cut off at SS7. After 4.5 hours (cf. the somitogenesis period is 1.5 hours), the right OV showed elongation comparable to that of the normal left OV. The tip of the right OV closed through wound healing.

Fig. S8. Quantification of local tissue deformation.
A: Live imaging using two-photon microscopy allows tracing of each nucleus.
B, **C**, **D**: Examples of trajectories of neuroepithelial nuclei within the OVs when half the forebrain region was removed. Normal ventral tissue (**B**), normal dorsal tissue (**C**), and cyclopamine-treated ventral tissue (**D**). As mentioned in the main text, normal ventral tissue, which is influenced by SHH signaling, can elongate in the M-L direction even if the dorsal half is excised (although the opposite is not true), that is, there is a clear D-V asymmetry in self-organization ability. The self-organization ability of ventral tissue was lost under SHH inhibition (**D** and see also Fig. 4, **A** to **C**). **E**: Method to calculate local tissue deformation from nuclei trajectories (see Materials and Method for details). **F**: Quantitative comparison of cell shape and cell edge length at T=0 and T=4.5h (control: normal ventral tissues; cyclopamine: cyclopamine-treated ventral tissues).Cell shape polarity was quantified based on F-actin images (left). The orientation angle and polarity strength for the combined data from all samples for each case are shown. The cell size was evaluated by the distribution of edge length (right). The sample sizes for control-0h, control-4.5h, cyclopamine-0h, and cyclopamine-4.5h are 10, 6, 8 and 8, respectively. In both control and cyclopamine-treated cases, the changes in cell shape and size within the time period of interest were minor.

Fig. S9. Time-lapse imaging to observe changes in cell adjacency relationship.
A: Schematic diagrams of the experiment (modified from Fig. 3**A**). **B**: Examples of changes in cell adjacency relationships. The time lapse imaging interval was 3 min. **C**: Mathematical representation of the process of changes in adjacency. See Materials and Methods for details. **D**: A sample representing M-L elongation; the bottom panels show the positions of traced cells (colored dots) and adjacency relationship (black line segments). It should be noted that the observed tissue elongation was somewhat lower than that calculated based on nuclei tracking data because more intense laser power was needed to detect the membrane.

Fig. S10. Quantification of cellular movement in response to external tissue stress.
A: Schematic diagrams of stress loading test (modification of Fig. 5**A**). By changing the orientation of the tissue to be attached, the applied stress direction was changed (right). **B-E**: Examples of nuclei trajectories for 3 hours under external stress. The rightmost panels show nuclei net displacement.

Fig. S11. Mechanical simulations to calculate 3D patterns of tissue stress within the forebrain region.
A: Polygonal mesh used for finite element simulations to calculate stress patterns. The 3D polygonal models were obtained based on two-photon microscopy images. The images were smoothed to create shapes with L-R symmetry. **B**: Simulation settings (see also Materials and Methods).

Fig. S12. Directions of maximum principal stress under different pressure
Directions of maximum principal stress were calculated under the hydrostatic pressure of 1 (kPa) and 10 (kPa). The patterns of the directions were robust against the value of the pressure.

Legends for Movies S1 to S7

Movie S1. 3D time-lapse imaging of a ventral forebrain tissue with the dorsal-half removed under control conditions. Related to Figs. 3**F-H** and Fig. S8**B**. Images were acquired at 10 minutes interval. Green dots are tracked nuclei. The horizontal (or vertical) direction represents the M-L (or A-P) axis.

Movie S2. 3D time-lapse imaging of a dorsal forebrain tissue with the ventral-half removed under control conditions. Related to Figs. 3**F-H** and Fig. S8**C**. Images were acquired at 10 minutes interval. Green dots are tracked nuclei. The horizontal (or vertical) direction represents the M-L (or A-P) axis.

Movie S3. 3D time-lapse imaging of a ventral forebrain tissue with the dorsal-half removed under the treatment of cyclopamine. Related to Figs. 3**F-H** and Fig. S8**D**. Images were acquired at 10 minutes interval. Green dots are tracked nuclei. The horizontal (or vertical) direction represents the M-L (or A-P) axis.

Movie S4. 3D time-lapse imaging of a normal ventral forebrain tissue pasted on a PDMS stretch chamber in stress loading tests; under A-P stress. Related to Figs. 5**E-F** and Fig. S10**B**. Images were acquired at 5 minutes interval. Green dots are tracked nuclei. The horizontal (or vertical) direction represents the M-L (or A-P) axis.

Movie S5. 3D time-lapse imaging of a normal ventral forebrain tissue pasted on a PDMS stretch chamber in stress loading tests; under M-L stress. Related to Figs. 5**E-F** and Fig. S10**C**. Images were acquired at 5 minutes interval. Green dots are tracked nuclei. The horizontal (or vertical) direction represents the M-L (or A-P) axis.

Movie S6. 3D time-lapse imaging of a ventral forebrain tissue with cyclopamine treatment pasted on a PDMS stretch chamber in stress loading tests; under A-P stress. Related to Figs. 5**E-F** and Fig. S10**D**. Images were acquired at 5 minutes interval. Green dots are tracked nuclei. The horizontal (or vertical) direction represents the M-L (or A-P) axis.

Movie S7. 3D time-lapse imaging of a ventral forebrain tissue with cyclopamine treatment pasted on a PDMS stretch chamber in stress loading tests; under M-L stress. Related to Figs. 5**E-F** and Fig. S10**E**. Images were acquired at 5 minutes interval. Green dots are tracked nuclei. The horizontal (or vertical) direction represents the M-L (or A-P) axis.

Figure S1

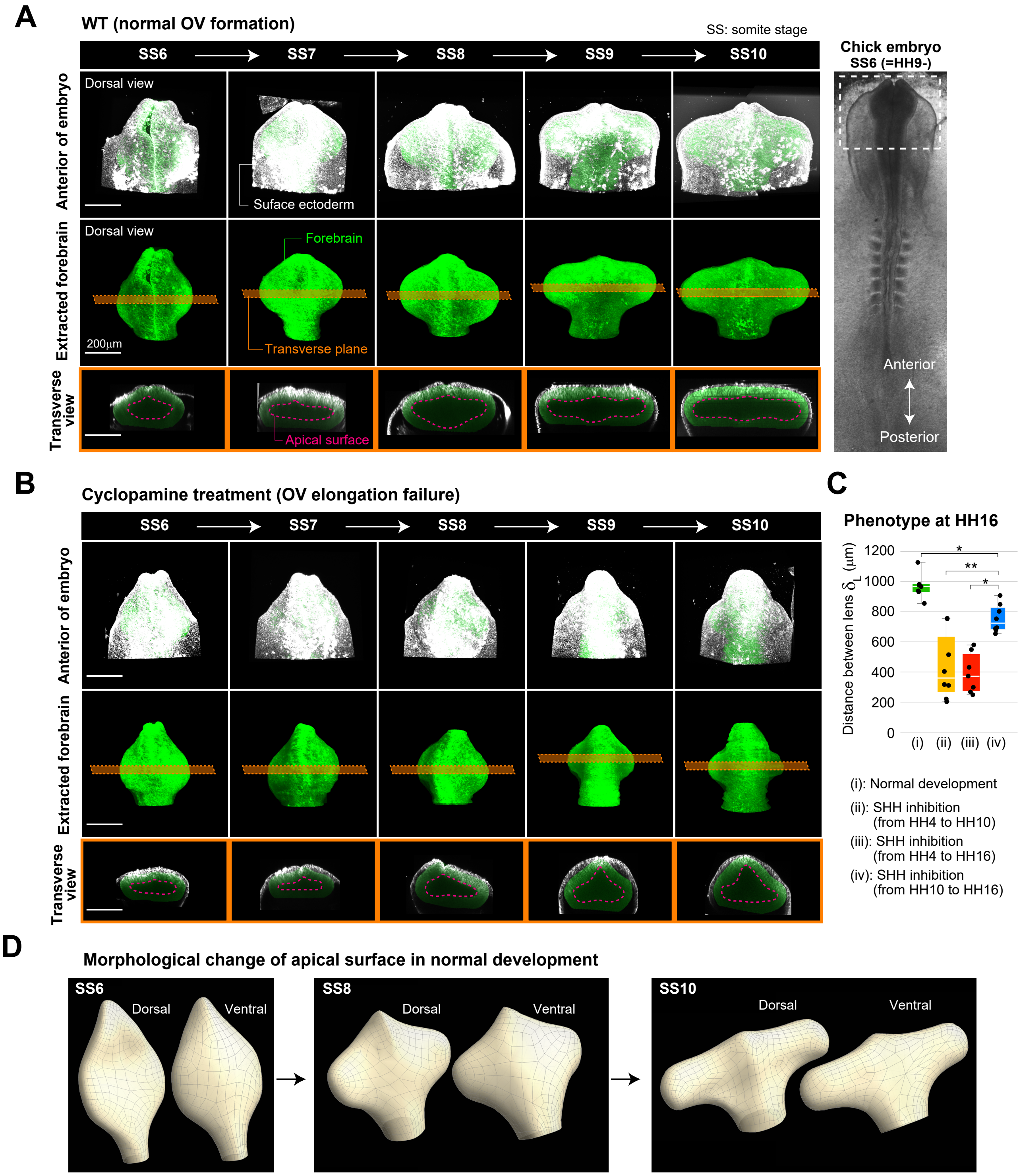
A
WT (normal OV formation)
SS: somite stage
SS6
SS7
SS8
SS9
SS10
Anterior of embryo
Dorsal view
Suface ectoderm
Extracted forebrain
Dorsal view
Forebrain
Transverse plane
200µm
Transverse view
Apical surface
Chick embryo
SS6 (=HH9-)
Anterior
Posterior
B
Cyclopamine treatment (OV elongation failure)
SS6
SS7
SS8
SS9
SS10
Anterior of embryo
Extracted forebrain
Transverse view
C
Phenotype at HH16
Distance between lens δL (µm)
1200
1000
800
600
400
200
0
(i)
(ii)
(iii)
(iv)
*
**
*
(i): Normal development
(ii): SHH inhibition (from HH4 to HH10)
(iii): SHH inhibition (from HH4 to HH16)
(iv): SHH inhibition (from HH10 to HH16)
D
Morphological change of apical surface in normal development
SS6
Dorsal
Ventral
SS8
Dorsal
Ventral
SS10
Dorsal
Ventral

Figure S2

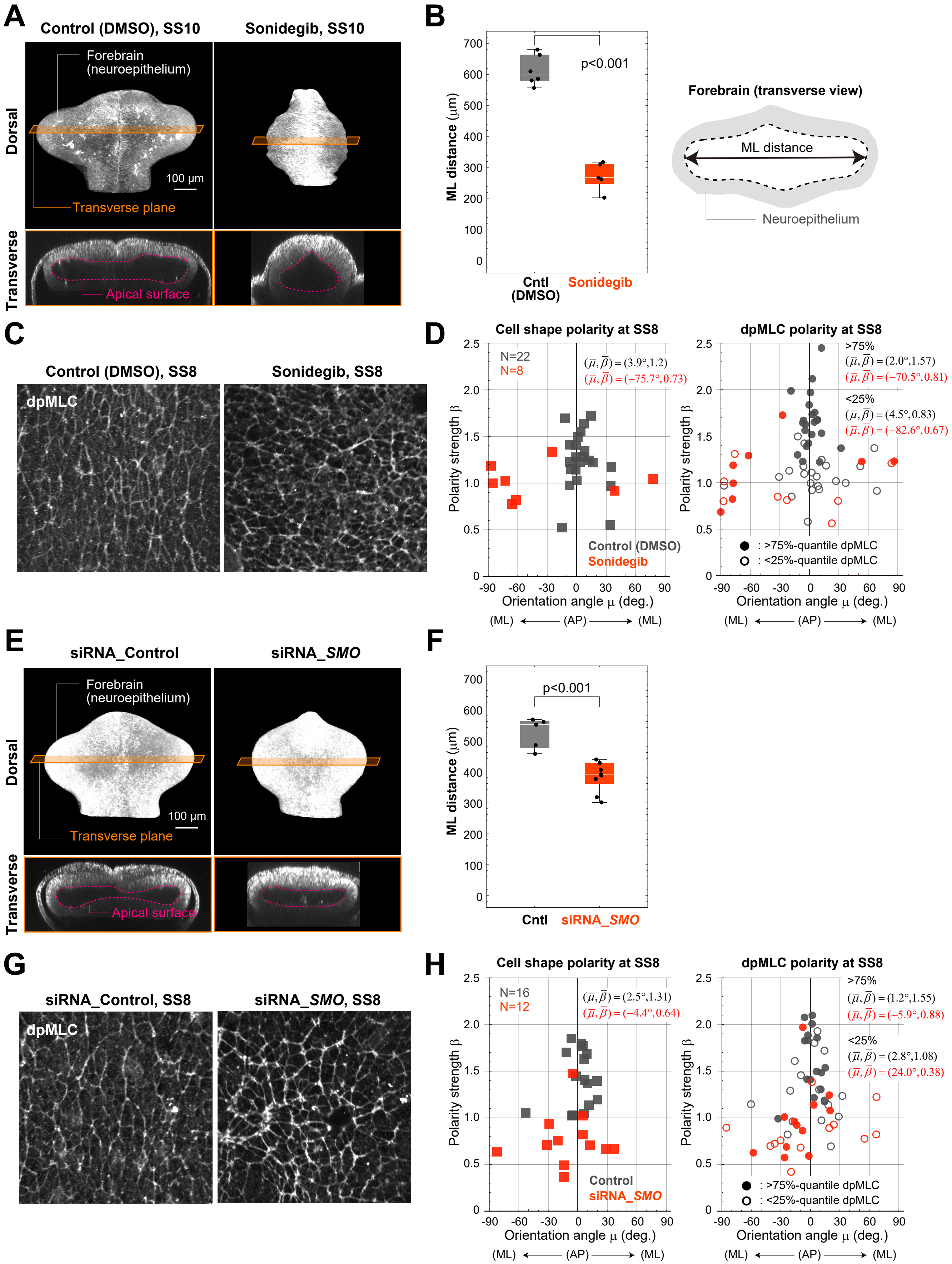
A
Control (DMSO), SS10
Sonidegib, SS10
Dorsal
Transverse
Forebrain
(neuroepithelium)
100 μm
Transverse plane
Apical surface
B
ML distance (μm)
p<0.001
Cntl
(DMSO)
Sonidegib
Forebrain (transverse view)
ML distance
Neuroepithelium
C
Control (DMSO), SS8
Sonidegib, SS8
dpMLC
D
Cell shape polarity at SS8
dpMLC polarity at SS8
N=22
N=8
Polarity strength β
Orientation angle μ (deg.)
(ML) (AP) (ML)
Control (DMSO)
Sonidegib
>75%
<25%
: >75%-quantile dpMLC
: <25%-quantile dpMLC
E
siRNA_Control
siRNA_*SMO*
Dorsal
Transverse
Forebrain
(neuroepithelium)
100 μm
Transverse plane
Apical surface
F
ML distance (μm)
p<0.001
Cntl
siRNA_*SMO*
G
siRNA_Control, SS8
siRNA_*SMO*, SS8
dpMLC
H
Cell shape polarity at SS8
dpMLC polarity at SS8
N=16
N=12
Polarity strength β
Orientation angle μ (deg.)
(ML) (AP) (ML)
Control
siRNA_*SMO*
>75%
<25%
: >75%-quantile dpMLC
: <25%-quantile dpMLC

Figure S3

Brief summary of our previous work [Ref. (11)]

A 3D morphological change in apical surface of neuroepithelium and cell trajectories

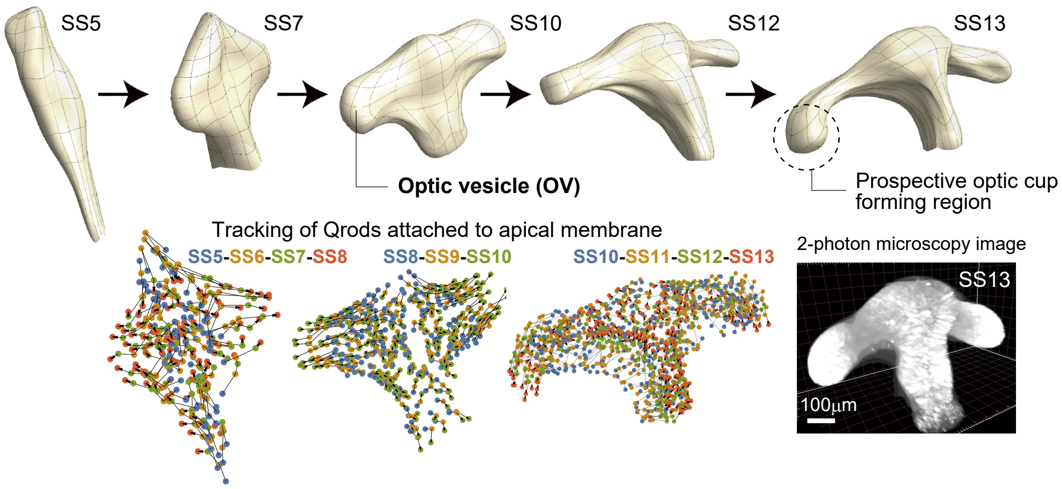

B Bayesian reconstruction of tissue deformation maps (normal development)

Examples of reconstructed spatio-temporal patterns of local tissue deformation

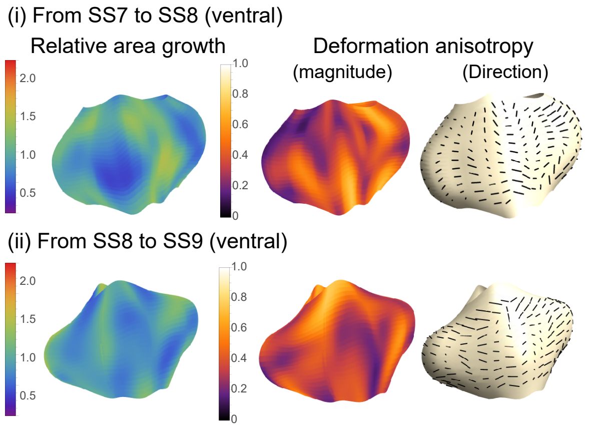

Main features of tissue-level dynamics

- No clear spatial-bias in area growth rate. (i.e., OV elongation is not driven by differential growth)
- Deformation anisotropy is high over the entire forebrain (not only the OV region), and its orientation is strongly biased to the ML direction.

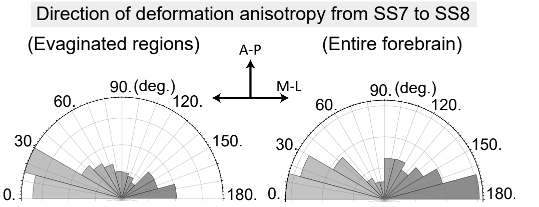

C Cellular behaviors during optic vesicle formation/elongation

- Cell proliferation is not necessary for OV elongation.

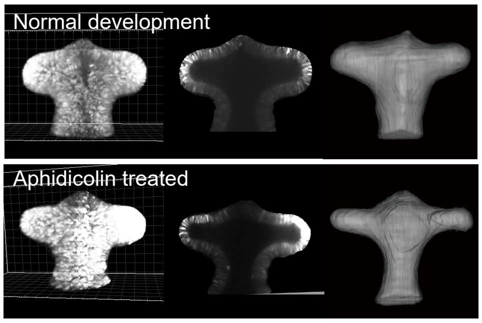

- Orientation of cell division is almost random.

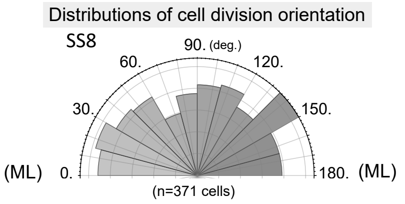

- Initially (~SS7), cells are randomly oriented or have a slight A-P bias and in the elongated OV, they are oriented in the AP direction, perpendicular to the direction of tissue deformation.

* The diagrams show the results for all data for ventral and dorsal cells.

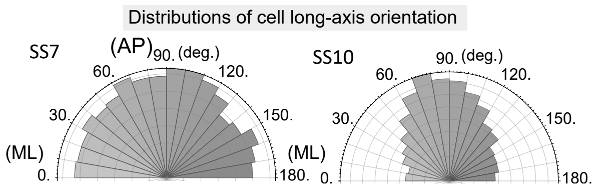

Conclusion

- Cell division (frequency, orientation) and cell shape/size change cannot explain ML-biased anisotropic tissue deformation observed during OV elongation.
- Directional cell rearrangement may achieve the observed anisotropic tissue deformation, although more detailed analysis on collective cell motion is needed.

Figure S4

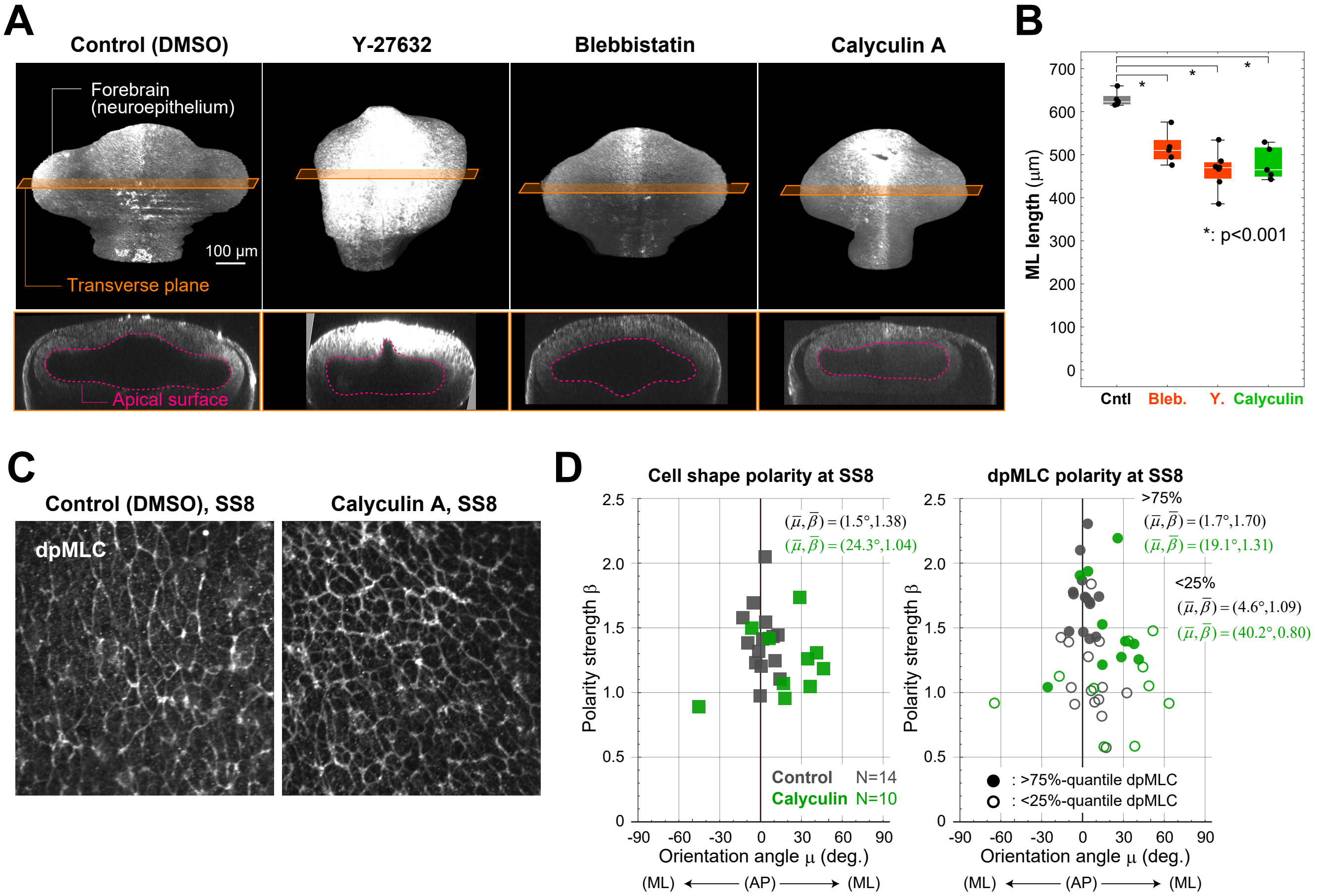

A
Control (DMSO)
Y-27632
Blebbistatin
Calyculin A
Forebrain (neuroepithelium)
100 μm
Transverse plane
Apical surface
B
ML length (μm)
*: p<0.001
Cntl
Bleb.
Y.
Calyculin
C
Control (DMSO), SS8
Calyculin A, SS8
dpMLC
D
Cell shape polarity at SS8
dpMLC polarity at SS8
Polarity strength β
Orientation angle μ (deg.)
(ML) (AP) (ML)
$(\bar{\mu},\bar{\beta})=(1.5°,1.38)$
$(\bar{\mu},\bar{\beta})=(24.3°,1.04)$
Control N=14
Calyculin N=10
>75%
$(\bar{\mu},\bar{\beta})=(1.7°,1.70)$
$(\bar{\mu},\bar{\beta})=(19.1°,1.31)$
<25%
$(\bar{\mu},\bar{\beta})=(4.6°,1.09)$
$(\bar{\mu},\bar{\beta})=(40.2°,0.80)$
: >75%-quantile dpMLC
: <25%-quantile dpMLC

Figure S5

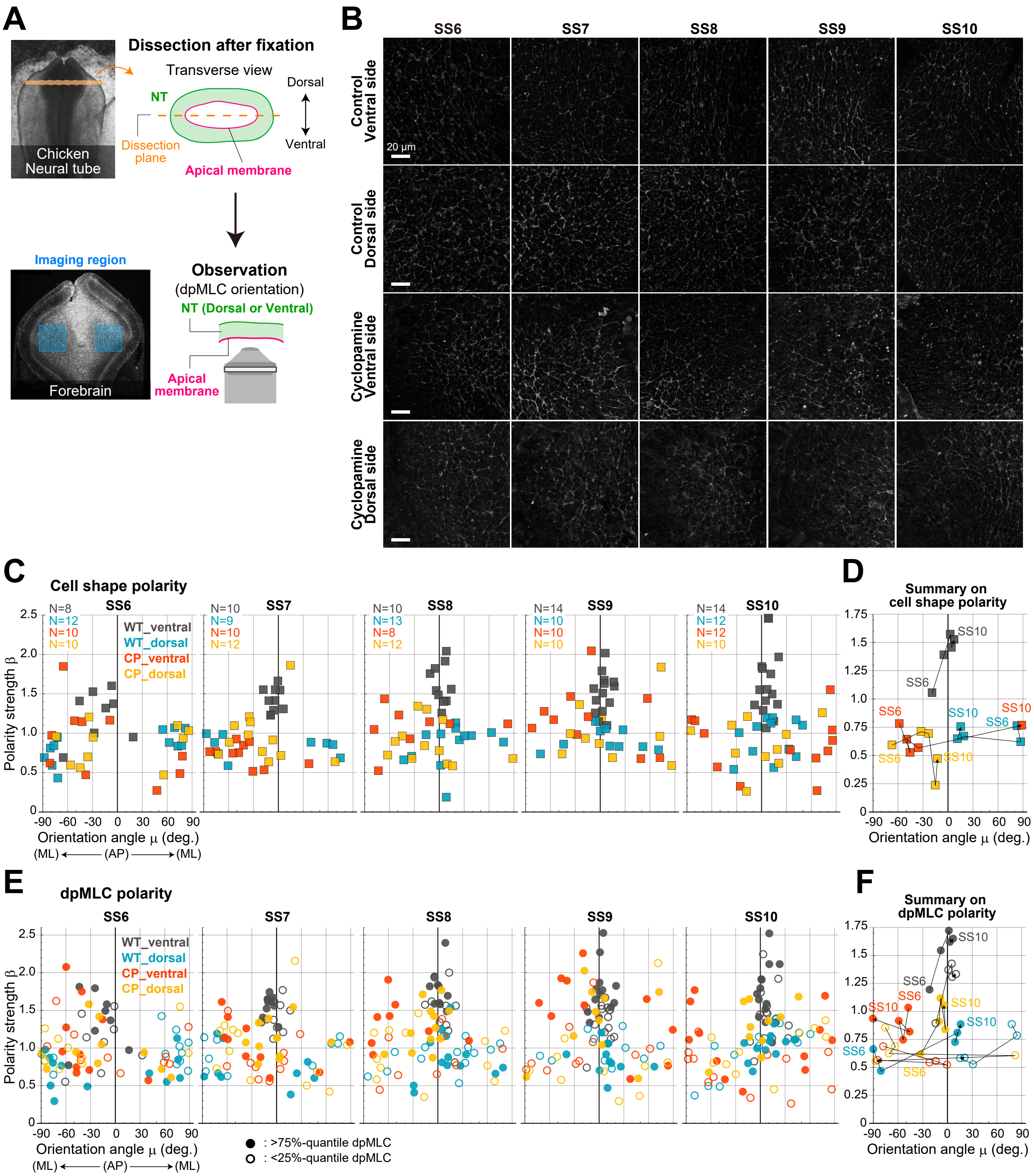
A
Dissection after fixation
Transverse view
Dorsal
Ventral
NT
Dissection plane
Apical membrane
Chicken Neural tube
Imaging region
Forebrain
Observation
(dpMLC orientation)
NT (Dorsal or Ventral)
Apical membrane
B
SS6
SS7
SS8
SS9
SS10
Control Ventral side
Control Dorsal side
Cyclopamine Ventral side
Cyclopamine Dorsal side
20 µm
C
Cell shape polarity
WT_ventral
WT_dorsal
CP_ventral
CP_dorsal
Polarity strength β
Orientation angle µ (deg.)
(ML) (AP) (ML)
D
Summary on cell shape polarity
E
dpMLC polarity
: >75%-quantile dpMLC
: <25%-quantile dpMLC
F
Summary on dpMLC polarity

Figure S6

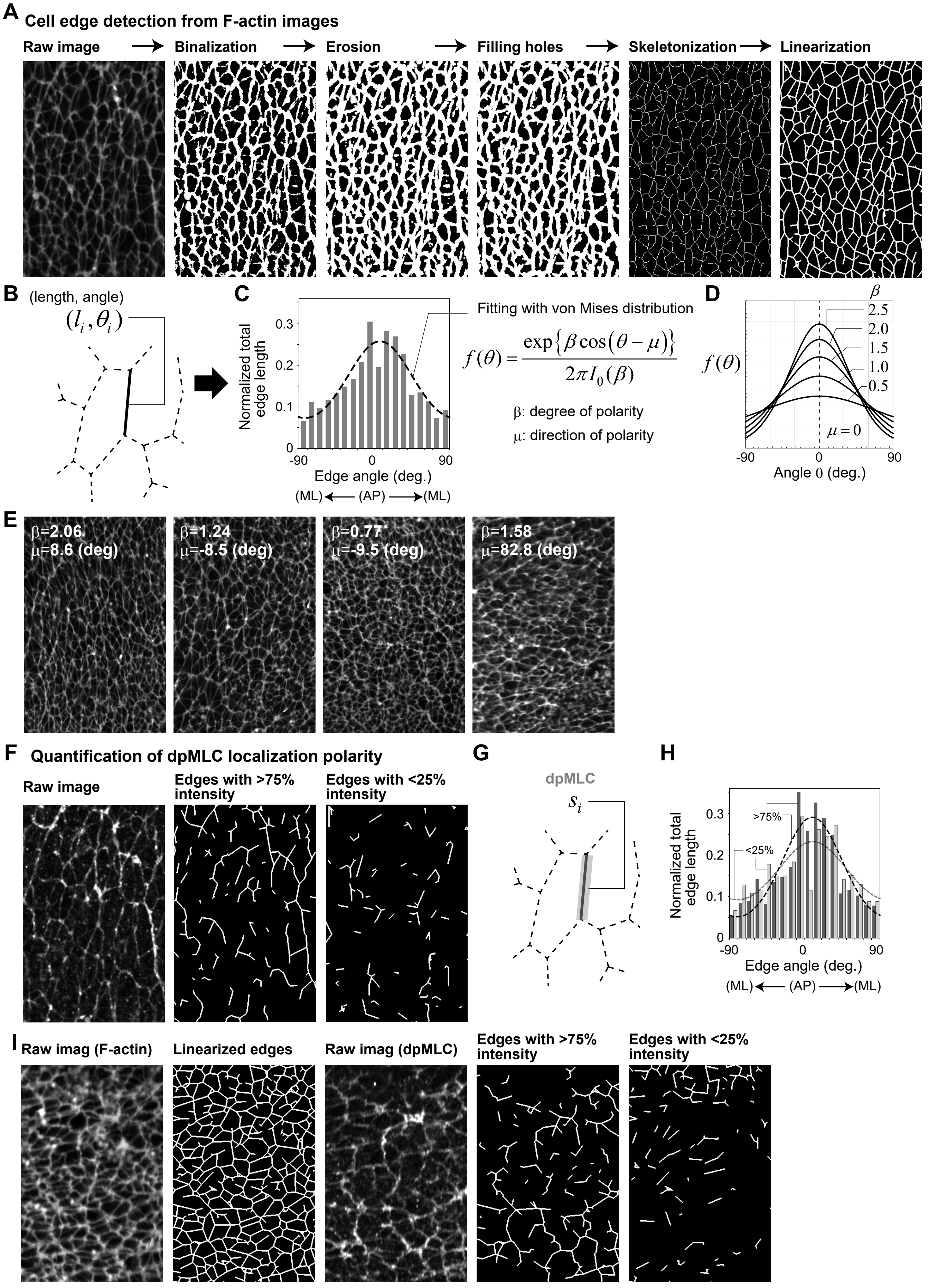

A Cell edge detection from F-actin images
Raw image
Binalization
Erosion
Filling holes
Skeletonization
Linearization
B (length, angle)
(l_i, θ_i)
C
Normalized total edge length
0.3
0.2
0.1
0
-90
0
90
Edge angle (deg.)
(ML) (AP) (ML)
Fitting with von Mises distribution
$f(\theta) = \frac{\exp\{\beta \cos(\theta - \mu)\}}{2\pi I_0(\beta)}$
β: degree of polarity
μ: direction of polarity
D
β
2.5
2.0
1.5
1.0
0.5
$f(\theta)$
$\mu = 0$
Angle θ (deg.)
E
β=2.06
μ=8.6 (deg)
β=1.24
μ=-8.5 (deg)
β=0.77
μ=-9.5 (deg)
β=1.58
μ=82.8 (deg)
F Quantification of dpMLC localization polarity
Raw image
Edges with >75% intensity
Edges with <25% intensity
G
dpMLC
s_i
H
Normalized total edge length
>75%
<25%
Edge angle (deg.)
(ML) (AP) (ML)
I
Raw imag (F-actin)
Linearized edges
Raw imag (dpMLC)
Edges with >75% intensity
Edges with <25% intensity

Figure S7

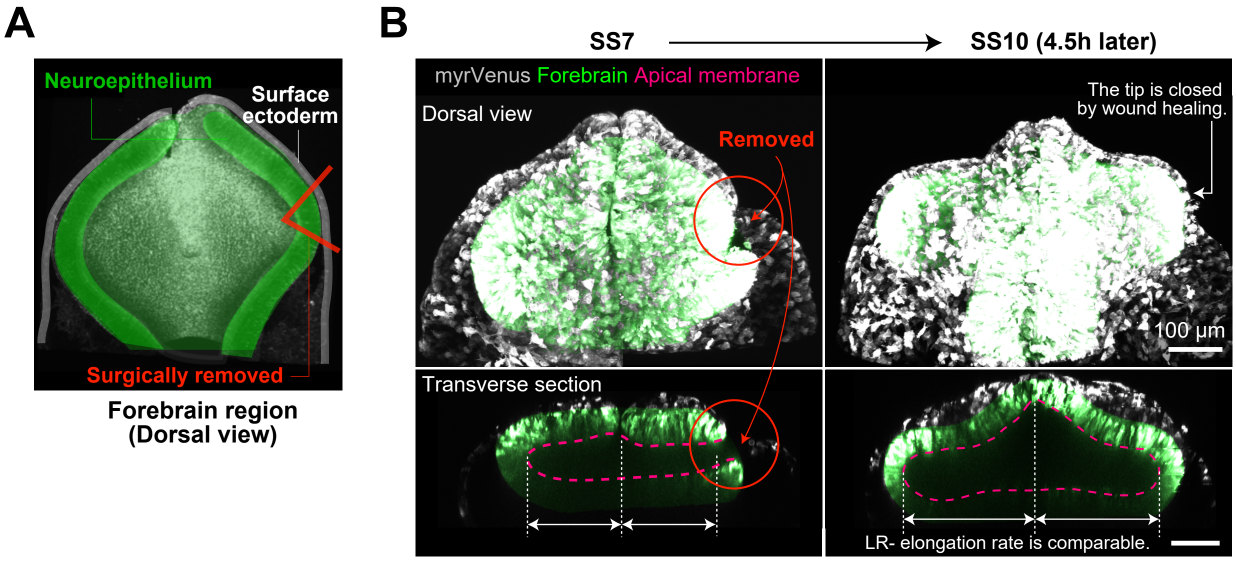
A
Neuroepithelium
Surface ectoderm
Surgically removed
Forebrain region
(Dorsal view)
B
SS7
SS10 (4.5h later)
myrVenus Forebrain Apical membrane
Dorsal view
Removed
The tip is closed by wound healing.
100 µm
Transverse section
LR- elongation rate is comparable.

Figure S8

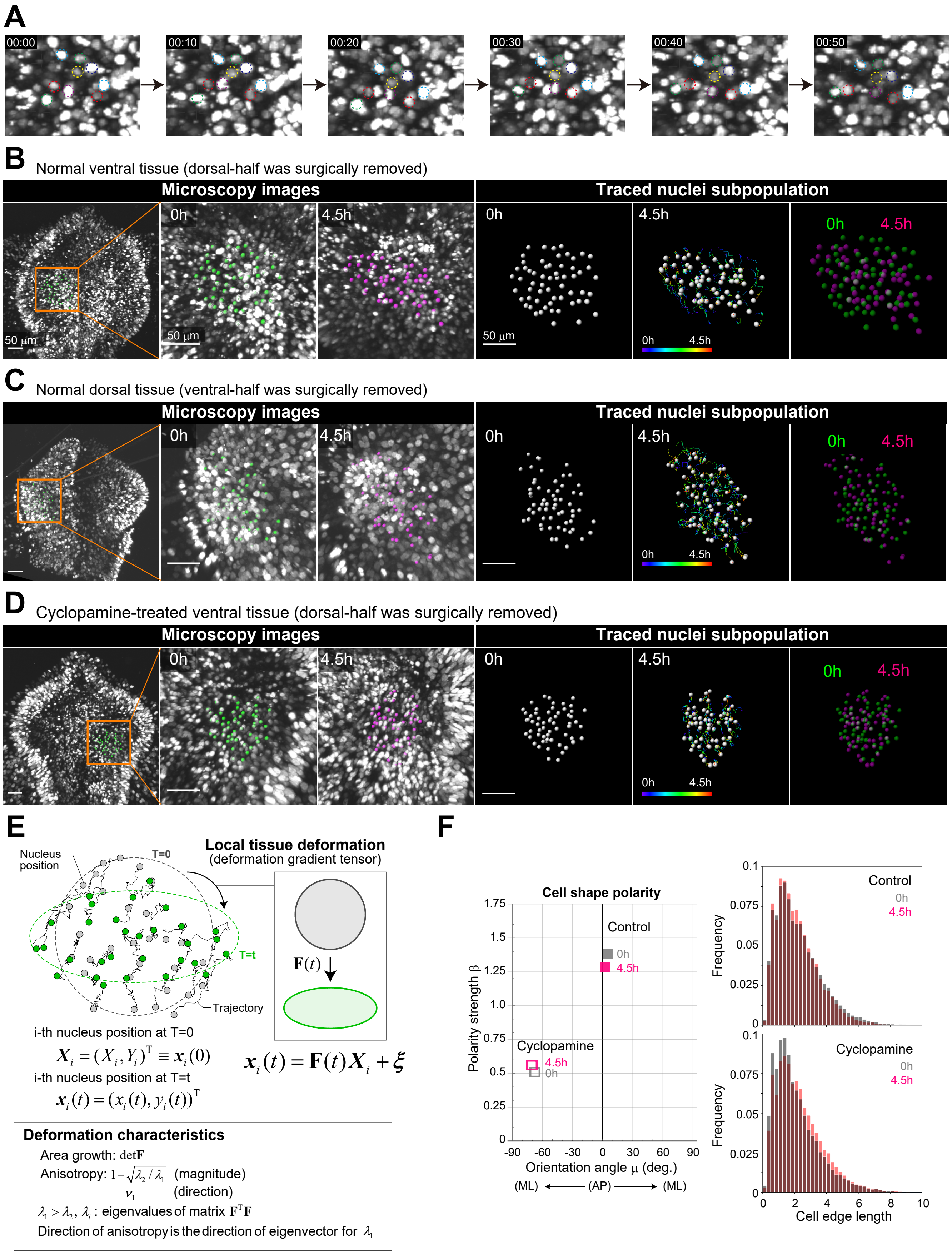

A
00:00
00:10
00:20
00:30
00:40
00:50
B
Normal ventral tissue (dorsal-half was surgically removed)
Microscopy images
Traced nuclei subpopulation
0h
4.5h
50 μm
C
Normal dorsal tissue (ventral-half was surgically removed)
D
Cyclopamine-treated ventral tissue (dorsal-half was surgically removed)
E
Nucleus position
T=0
T=t
Trajectory
Local tissue deformation
(deformation gradient tensor)
F(t)
i-th nucleus position at T=0
$X_i = (X_i, Y_i)^T \equiv x_i(0)$
i-th nucleus position at T=t
$x_i(t) = (x_i(t), y_i(t))^T$
$x_i(t) = F(t)X_i + \xi$
Deformation characteristics
Area growth: detF
Anisotropy: $1-\sqrt{\lambda_2/\lambda_1}$ (magnitude)
v_1 (direction)
$\lambda_1 > \lambda_2$, λ_i : eigenvalues of matrix $F^T F$
Direction of anisotropy is the direction of eigenvector for λ_1
F
Cell shape polarity
Control
Cyclopamine
Polarity strength β
Orientation angle μ (deg.)
(ML) (AP) (ML)
Frequency
Cell edge length

Figure S9

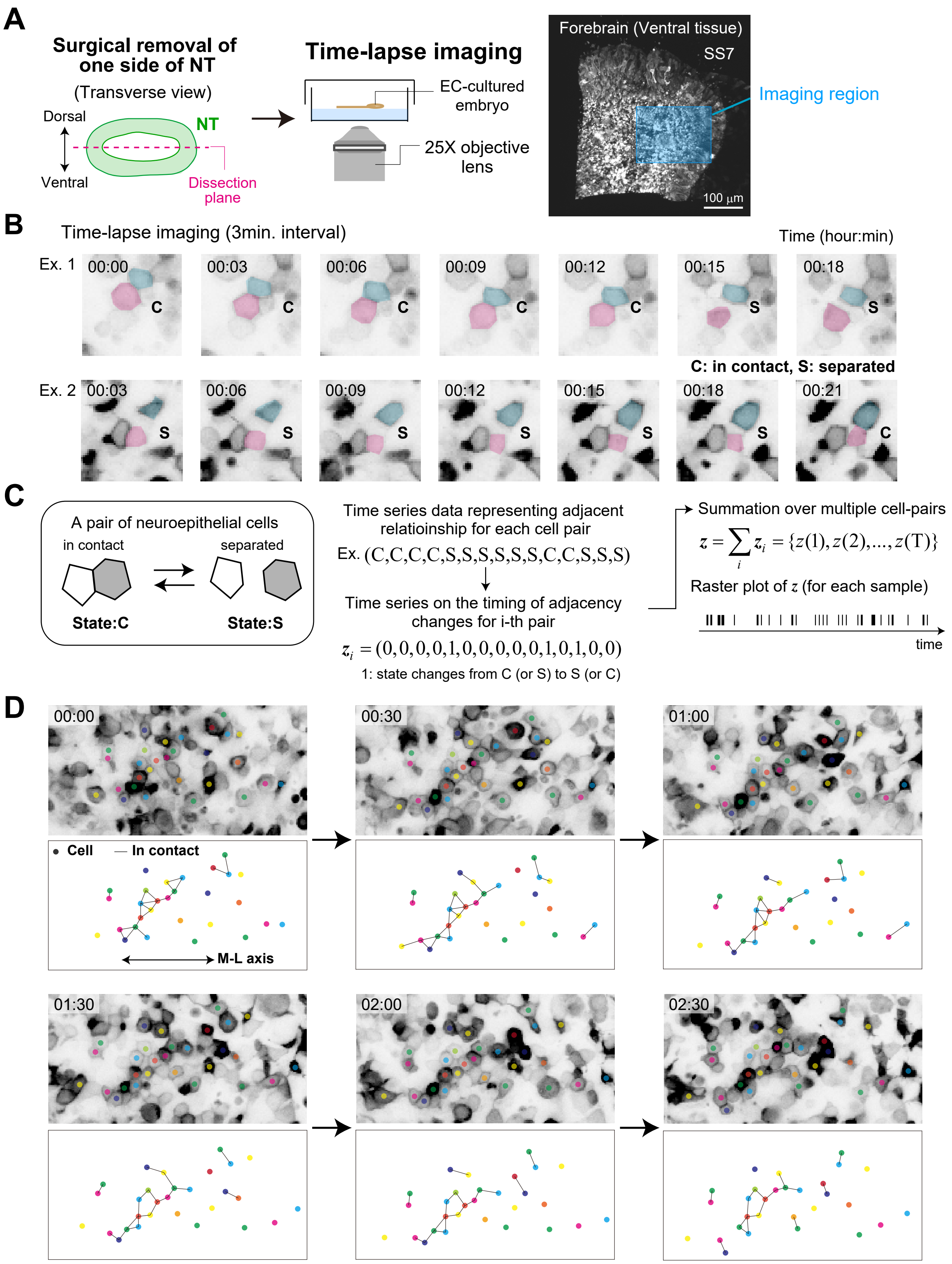
A
Surgical removal of one side of NT
(Transverse view)
Dorsal
Ventral
NT
Dissection plane
Time-lapse imaging
EC-cultured embryo
25X objective lens
Forebrain (Ventral tissue)
SS7
Imaging region
100 µm
B
Time-lapse imaging (3min. interval)
Time (hour:min)
Ex. 1
00:00 C
00:03 C
00:06 C
00:09 C
00:12 C
00:15 S
00:18 S
C: in contact, S: separated
Ex. 2
00:03 S
00:06 S
00:09 S
00:12 S
00:15 S
00:18 S
00:21 C
C
A pair of neuroepithelial cells
in contact
separated
State:C
State:S
Time series data representing adjacent relatioinship for each cell pair
Ex. (C,C,C,C,S,S,S,S,S,S,C,C,S,S,S)
Time series on the timing of adjacency changes for i-th pair
$z_i = (0,0,0,0,1,0,0,0,0,0,1,0,1,0,0)$
1: state changes from C (or S) to S (or C)
Summation over multiple cell-pairs
$z = \sum_i z_i = \{z(1), z(2), ..., z(T)\}$
Raster plot of z (for each sample)
time
D
00:00
00:30
01:00
Cell
In contact
M-L axis
01:30
02:00
02:30

Figure S10

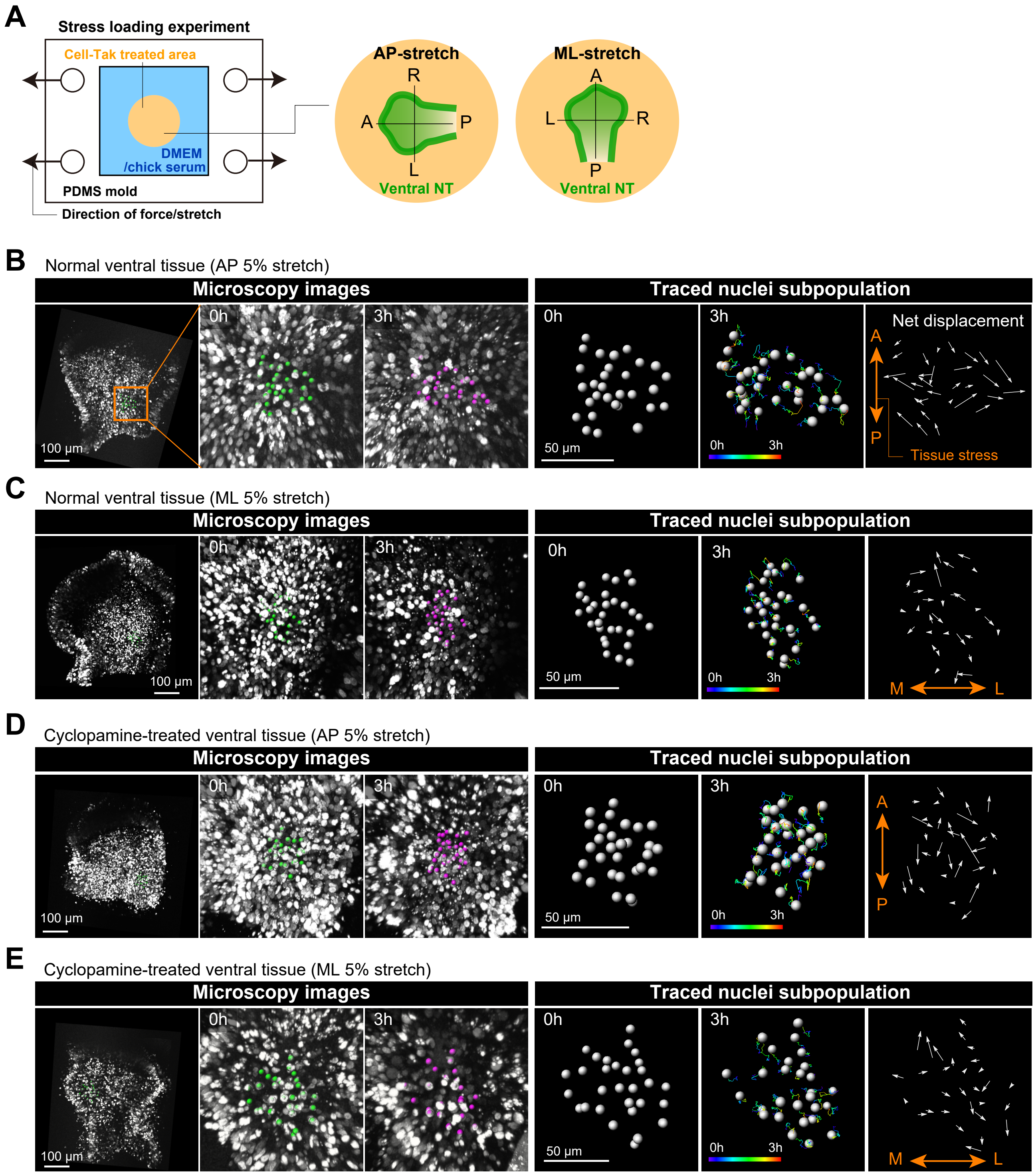
A
Stress loading experiment
Cell-Tak treated area
DMEM /chick serum
PDMS mold
Direction of force/stretch
AP-stretch
R
A
P
L
Ventral NT
ML-stretch
A
L
R
P
Ventral NT
B
Normal ventral tissue (AP 5% stretch)
Microscopy images
Traced nuclei subpopulation
0h
3h
Net displacement
Tissue stress
100 µm
50 µm
C
Normal ventral tissue (ML 5% stretch)
Microscopy images
Traced nuclei subpopulation
M
L
D
Cyclopamine-treated ventral tissue (AP 5% stretch)
Microscopy images
Traced nuclei subpopulation
E
Cyclopamine-treated ventral tissue (ML 5% stretch)
Microscopy images
Traced nuclei subpopulation

Figure S11

A

Hexahedral mesh for finite element simulation (3D, thick-walled)

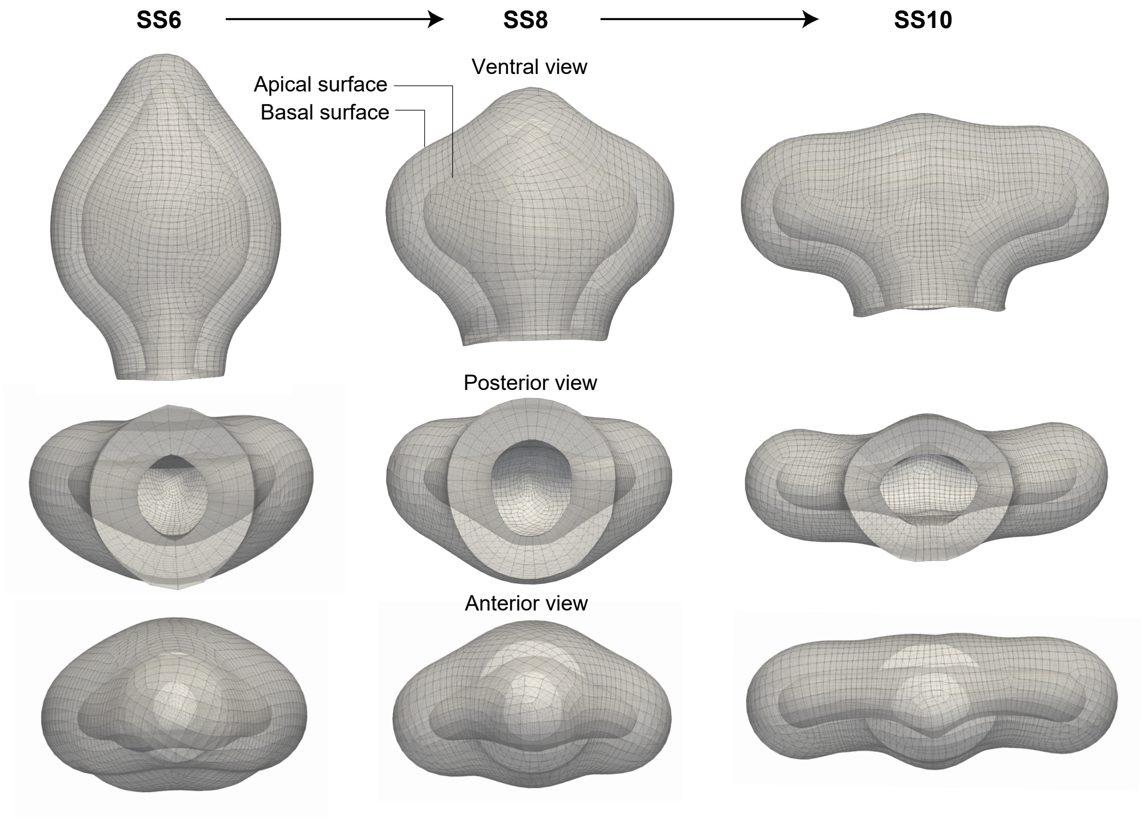

B

Model assumptions

- (Slightly) compressible hyperelastic material
- Strain energy function Ψ:

$$\Psi(J,\bar{I}_1)=\frac{\kappa}{2}(J-1)^2+c(\bar{I}_1-3)$$

$$J=\det\mathbf{F} \quad (\mathbf{F}\text{: deformation gradient tensor})$$

$$\bar{I}_1=\mathrm{tr}(\bar{\mathbf{F}}^{\mathrm{T}}\bar{\mathbf{F}})$$

$$\bar{\mathbf{F}}=J^{-1/3}\mathbf{F}$$

$$\kappa=1.0\times10^4\ [\mathrm{kPa}]$$

$$c=1.0\times10^2\ [\mathrm{kPa}]$$

- Small hydrostatic pressure
- Posterior ends can move only in the transverse plane.

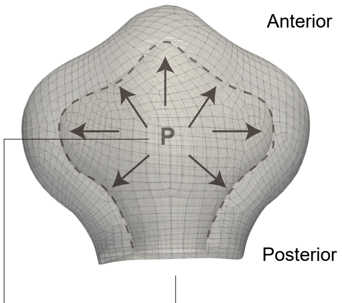

Figure S12

Directions of maximum principal stress under different pressure

**Hydrostatic pressure**	**Developmental stage** SS6	SS7	SS8	SS10
p=1 [kPa]	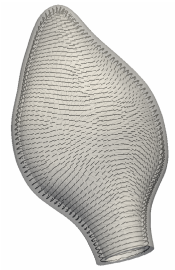	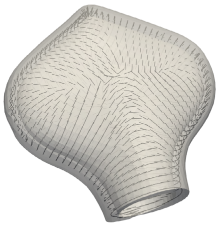	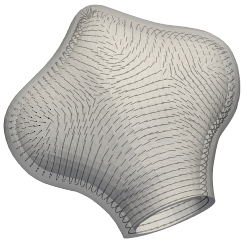	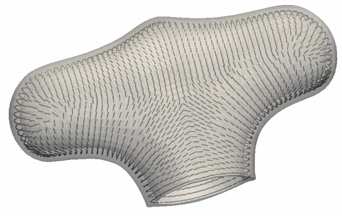
p=10 [kPa]	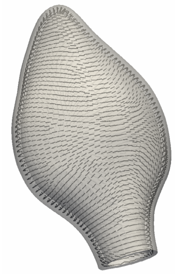	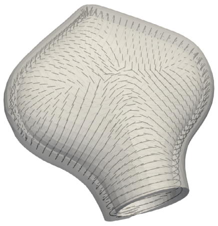	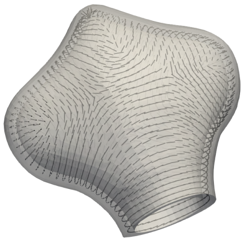	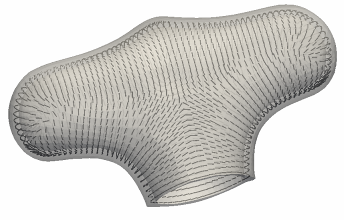

Table S1: Primers for plasmid construction

Gene	Forward Primer Sequence	Reverse Primer Sequence
H2B-Venus	AAGCTTATGCCAGAGCCAGCGAAGTC	TCTAGATTACTTGTACAGCTCGTCC

Table S2: RT-qPCR primers

Gene	Forward Primer Sequence	Reverse Primer Sequence
SHH	GATGAAGAGAACACGGGAGC	GATTCCTCGGAGTGATGGCC
PTCH1	CCAACGTAGAGGAGCTGTGG	AGCTGGGGGTTAAACATGGC
FOXA2	ACAAGATGCTGACGCTGAGC	CCTTTGCCCGGCTTGTCTGG
MED21	TACAGGATGCGGTGAACTCG	ATGTTGCTGAAGGAAGCTGG
